# Carbonate-Enhanced Photoelectrochemical Corrosion Limits the CO_2_ Reduction Reactivity on CuFeO_2_ Delafossite Photocathodes

**DOI:** 10.1021/acs.jpcc.5c08145

**Published:** 2026-03-26

**Authors:** Piyush Anil Kumar Sharma, Jaclyn A. Rebstock, Ting-Rong Ko, Emma Pollock, Jeffersson Feutseu, Julia C. Lam, Patrick M. Woodward, L. Robert Baker

**Affiliations:** Department of Chemistry and Biochemistry, 2647 The Ohio State University, Columbus, Ohio 43210, United States

## Abstract

CuFeO_2_ delafossite materials have been researched for their promising photoactivity for CO_2_ reduction (CO_2_R) due to their intrinsic p-type conductivity. However, its practical application is limited by its poor stability and low photocurrent densities. In this work, we investigated the mechanistic origin of CuFeO_2_ degradation under CO_2_R conditions. Through photoelectrochemical measurements combined with ex situ X-ray photoelectron spectroscopy and in situ surface-enhanced Raman spectroscopy, we show that CO_2_-saturated sodium bicarbonate electrolytes enhance photoelectrochemical corrosion by facilitating iron leaching from the catalyst. Systematic control experiments reveal that this instability is not governed solely by thermodynamic surface stability but arises from a nonequilibrium interfacial speciation of CO_2_, bicarbonate, and carbonate. The presence of carbonate species at the catalyst interface facilitates iron­(II) complexation and degrades the CuFeO_2_ surface. These findings establish carbonate-driven photoelectrochemical corrosion as a key degradation pathway for CuFeO_2_ and underscore the importance of speciation at the interface-electrolyte in dictating the long-term performance of a catalyst for CO_2_R.

## Graphical abstract

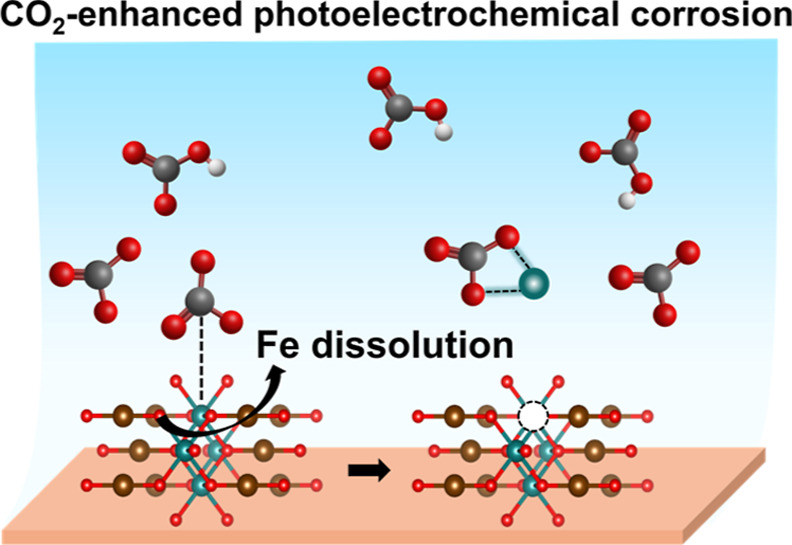

## Introduction

Metal oxide semiconductors have gained attention as a complementary class of catalysts for CO_2_ reduction (CO_2_R) catalysis. Oxide-based materials are often earth-abundant and are capable of integrating light absorption with catalytic functionality, making them suitable for photoelectrocatalysis.^1–4^ Among this class of metal oxide semiconductors, delafossite compounds (ABO_2_), where A is a monovalent transition metal cation and B is a trivalent cation, have recently attracted significant interest for solar energy harvesting as photoelectrocatalysts. Delafossite compounds have a unique layered structured composed of linearly coordinated A-site cations and edge-shared BO_6_ octahedra, which yields rich electronic transport properties.^5,6^ Among the delafossite family, copper iron oxide (CuFeO_2_) has emerged as a promising candidate owing to its earth-abundance, intrinsic p-type conductivity, and appropriate band gap, making it a potentially effective photocathode.^7–9^ Time-resolved X-ray and XUV spectroscopy studies have shown that this layered structure enhances photocatalytic activity by facilitating rapid, interlayer charge separation.^10,11^ Consistent with this observation, reports have shown its activity toward both HER and CO_2_R.^12–14^ Despite having these promising features, some key limitations have hindered CuFeO_2_ from broader applications. It suffers from low current densities and long-term instability.^11,15,16^ In a previous report, we have specifically shown that CuFeO_2_ suffers from surface instability due to leaching of iron into the electrolyte during photoelectrochemistry.^17^ As a result, the measured catalytic activity reflects not only the intrinsic property of CuFeO_2_ but also additional contributions arising from its degradation, making it difficult to isolate the true activity of the material. These results underscore a significant concern for CuFeO_2_ photocathodes: while they may have appealing properties, their stability under reaction conditions is poor, and efforts to improve their stability are hindered by a lack of mechanistic understanding regarding the degradation mechanism.

In this research, we address this question by investigating the mechanistic origin of iron leaching in CuFeO_2_ under CO_2_R reaction conditions. Through carefully designed photoelectrochemical experiments combined with ex situ XPS and in situ surface-enhanced Raman spectroscopy (SERS), we investigated the origin of the instability, with particular focus on CO_2_-saturated bicarbonate electrolytes due to their relevance for CO_2_R. From these studies, we find that the nonequilibrium CO_2_/bicarbonate/carbonate speciation at the CuFeO_2_ catalyst surface during Faradaic CO_2_R contributes significantly to iron dissolution, highlighting that in addition to the thermodynamic limits of CuFeO_2_ stability, one must further consider the kinetics of photoelectrochemical corrosion, which depend strongly on the concentration gradients that form when electrochemical current drives the interface away from equilibrium. By systematically probing this mechanism, our work provides insight into the degradation pathways that affect CuFeO_2_ stability when it is used as a photocathode for CO_2_R.

## Results and Discussion

### Photoelectrochemical Current Enhancement under CO_2_R Conditions

Details of the synthesis and film deposition are described in the Supporting Information, but a brief description of the catalyst preparation method is provided here. We begin with phase pure CuFeO_2_ prepared by heating stoichiometric mixtures of Cu_2_O and Fe_2_O_3_ at 1000 °C under flowing argon. To prepare the catalytic films, an ink slurry is prepared by mixing the CuFeO_2_ powder in an isopropyl alcohol/water/Nafion mixture. Aliquots of the ink slurry were drop casted onto FTO substrates under heat followed by annealing in inert gas at 550 °C for 1 h, resulting in transparent, thin CuFeO_2_ films. A more detailed surface characterization of the resulting porous CuFeO_2_ films is provided in the Supporting Information.

Linear sweep voltammetry (LSV) was performed on the prepared CuFeO_2_ films under CO_2_ and N_2_ purging to understand the performance and stability of the CuFeO_2_ photocathode in the presence and absence of CO_2_. As prepared, these catalysts display no photocurrent, meaning that no change in current is observed in the presence/absence of illumination (see Figure S2 in the Supporting Information). However, annealing the catalysts at 550 °C for 1 hour in air results in formation of Cu vacancies, which serve as a p-type dopant resulting in measurable photocurrents consistent with previous reports.^15,16,18^ Characterization of the air-annealed CuFeO_2_ catalysts is provided in Figures S3 and S4 of the Supporting Information. LSVs of these catalysts were collected in 0.1 M NaHCO_3_ during chopped white light illumination shown in Figure 1. All potentials in this work are reported with respect to RHE. Upon CO_2_ purging, the photocurrent nearly doubles at +0.22 V from 32 μA/cm^2^ under N_2_ purging to 58 μA/cm^2^ under CO_2_ purging, while the corresponding dark current values were 95 μA/cm^2^ under CO_2_ and 66 μA/cm^2^ under N_2_.

**Figure 1:**
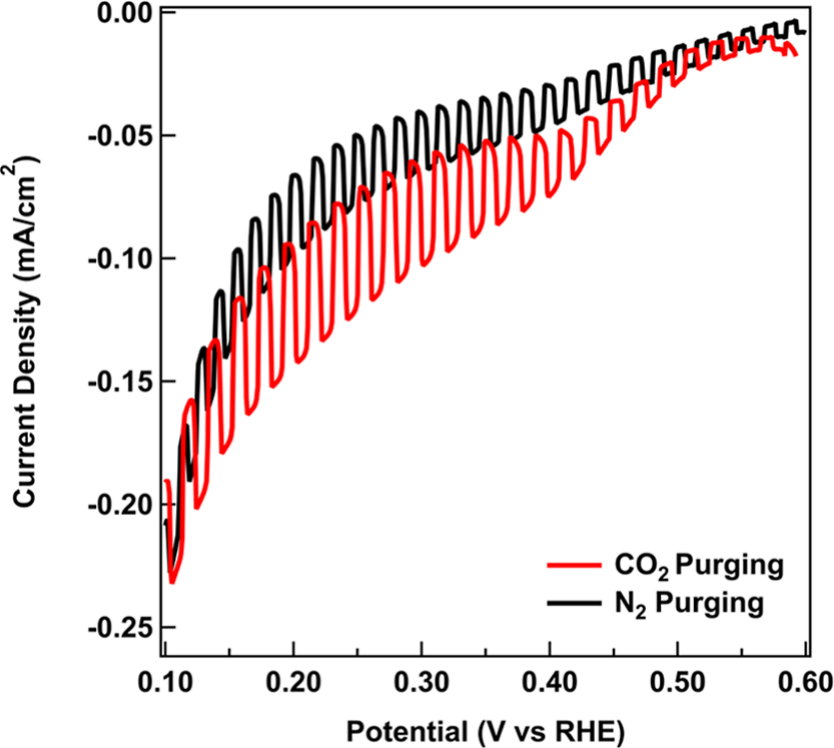
Linear sweep voltammograms in 0.1 M NaHCO_3_ under CO_2_ (red) and N_2_ (black) purging under chopped light illumination at 0.5 Hz.

CO_2_ purging decreases the pH of the solution from 8.1 to 6.9, indicating that the increased current could be due to the increased activity for the H_2_ evolution reaction (HER) in the slightly more acidic, CO_2_-purged electrolyte. However, we cannot conclude that this is the primary reason for the enhanced current because the increased thermodynamic driving force for HER is accounted for when placing both sets of data on the RHE scale. One alternate interpretation of these data is that CuFeO_2_ shows enhanced activity for CO_2_R compared to HER, which could explain why the photocurrent doubles in the presence of dissolved CO_2_. Unfortunately, we found that this is not the case. Using head space sampling by gas chromatography and nuclear magnetic resonance (NMR) measurements of the liquid phase, both with excellent detection limits, we do not observe any detectable formation of CO_2_R products. Note this is different than previous reports, where measurable yields of CO_2_R products have been reported.^13,15,19–21^ However, in those cases, mixed phase CuO/CuFeO_2_ catalysts were employed, while in this study we focus on the performance of phase pure CuFeO_2_. From this result, we hypothesize that the increase in photocurrent for phase pure CuFeO_2_ is not a result of CO_2_R activity but is instead due to enhanced photoelectrochemical corrosion of the CuFeO_2_ catalyst under CO_2_R reaction conditions. To confirm that this is the case, we compare the XPS of pre- and post-reaction catalysts.

### CuFeO_2_ Instability and the Role of CO_2_ in Iron Leaching

To investigate the iron leaching process, we performed XPS to quantify the presence of copper and iron within the CuFeO_2_ film before and after electrolysis. Detailed fitting procedures and quantification methods for XPS spectral analysis are provided in the Supporting Information. The electrolysis was run on the catalyst for 7 min under an applied bias. Figure 2 shows XPS measurements that were collected on the as-prepared CuFeO_2_ catalyst (Figure 2a) and ex situ after electrolysis under N_2_-saturated 0.1 M NaHCO_3_ at +0.33 V vs RHE (Figure 2b) and CO_2_-saturated 0.1 M NaHCO_3_ at +0.22 V vs RHE (Figure 2c) with constant light illumination. The Fe 2p scan of the as-prepared film shows the characteristics iron­(III) doublet due to spin-orbit splitting at 711.6 and 725.4 eV. In the spectra, the Fe 2p_3/2_ and Fe 2p_1/2_ peaks could be resolved into two peaks derived from the strong *j*–*j* coupling within the Fe 2p_3/2_ and Fe 2p_1/2_ states of iron­(III),^10,22^ although the Fe 2p peak is partially convoluted with the Sn 3p signal (shown in gray) from the FTO substrate, due to the thin nature of the electrically insulating CuFeO_2_ catalyst.^23,24^ The Cu 2p XPS spectra show a doublet peak located at 933.9 and 953.7 eV attributed to Cu 2p_3/2_ and Cu 2p_1/2_ spin–orbit components for copper­(II). The shakeup satellite features of copper­(II) confirming its presence are also present at 941.2, 943.6, and 962.0 eV. Meanwhile, the doublet binding energy of Cu 2p_3/2_ at 932.9 eV and Cu 2p_1/2_ at 952.5 eV can be assigned to the copper­(I) state.^25^ Although phase-pure CuFeO_2_ is expected to show only copper­(I), photocurrent is only observed when CuFeO_2_ catalysts are annealed in air (see Figure S2 of the Supporting Information), which introduces surface copper vacancies that serve as p-type dopants and manifest as copper­(II) in the XPS spectrum, as we have described in detail previously.^17^ However, we also find the formation of a small fraction of CuO postannealing as confirmed by XRD and Raman spectroscopy (see Figures S3 and S4 of the Supporting Information). Accordingly, this catalyst is best described as primarily CuFeO_2_ with surface copper vacancies introduced as a p-type dopant.

**Figure 2:**
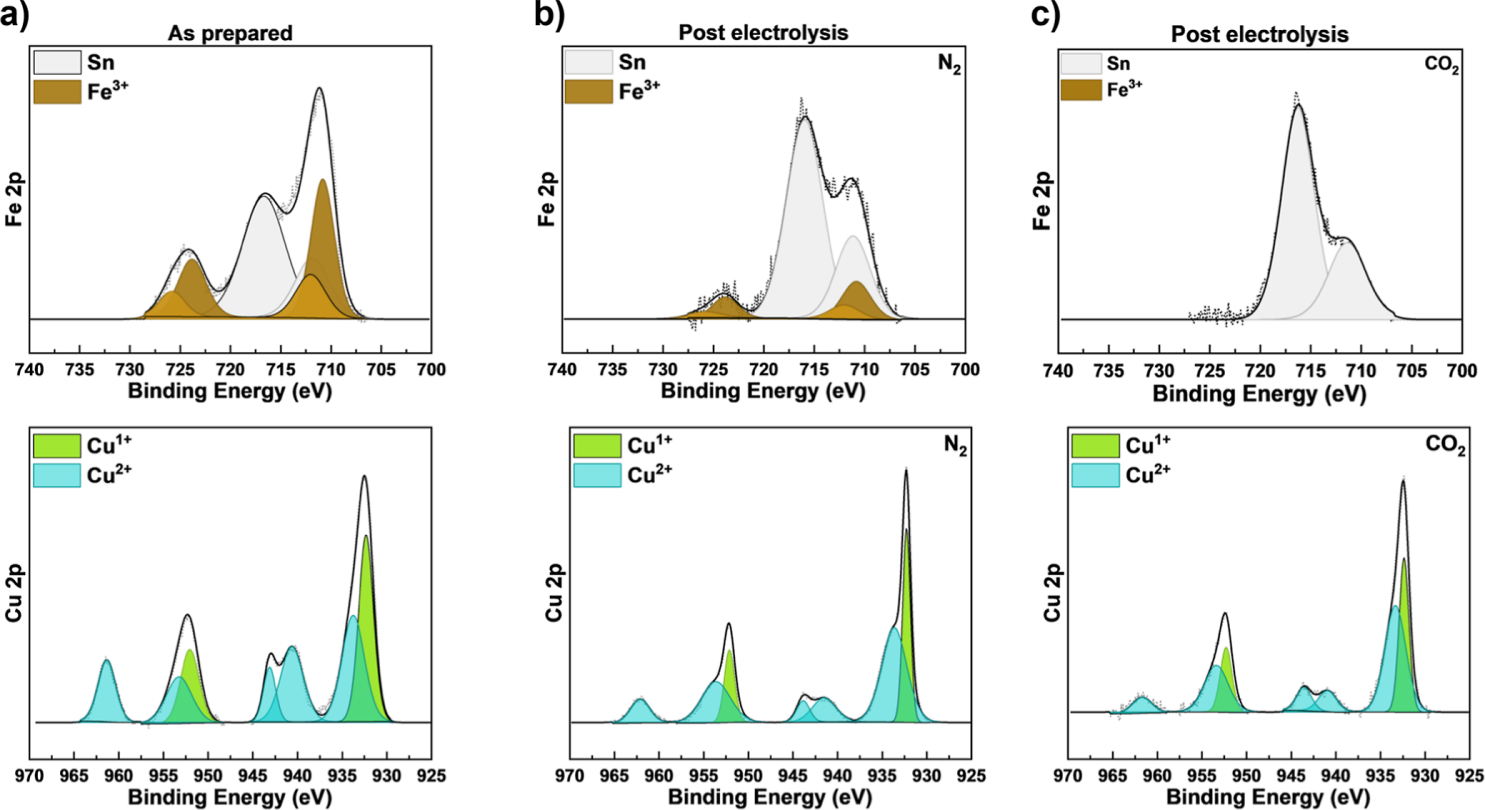
XPS of the (a) as-prepared CuFeO_2_ film and (b,c) post-electrolysis films. Electrolysis was run in CO_2_-saturated 0.1 M NaHCO_3_ at +0.22 V vs RHE and N_2_-saturated 0.1 M NaHCO_3_ at +0.33 V vs RHE under constant light illumination.

XPS analysis of the as-prepared film shows peak area percentages of 35% iron­(III) and 65% copper, with 55% from copper­(II) and the remaining 10% from copper­(I). The larger peak areas for copper compared to iron are a result of the roughly twice higher relative sensitivity factor of XPS for copper. Quantitative determination of elemental composition by XPS requires consideration of not only the relative sensitivity factors but also the energy-dependent and composition-dependent inelastic mean free path of the respective photoelectrons, making exact quantification difficult. However, comparison of the copper and iron peak areas before and after electrolysis in various conditions yields important insights into the photoelectrochemical corrosion mechanism. After electrolysis under N_2_ saturation, the iron­(III) atomic percent, which is the ratio of iron­(III) relative to the total amount of iron and copper, drops to 19% and the copper shows a reduction of the copper­(II) feature to 49%, indicating a partial reduction of copper and some leaching of iron under applied bias for the N_2_-purged electrolyte. In contrast, under CO_2_-saturated conditions, we observe a total loss of the iron signal. These findings support the hypothesis that the current measured during electrolysis is not due to CO_2_R but instead arises from enhanced photoelectrochemical corrosion of the CuFeO_2_ catalyst due to iron leaching in the presence of CO_2_.

In previous work, Ferri et al. analyzed the phase stability of bulk CuFeO_2_.^26^ Figure 3 represents the adapted version of the bulk Pourbaix diagram of CuFeO_2_ calculated using the experimental formation free energies for the Cu–Fe–O phases. This diagram maps the stability region of the catalyst as a function of pH and applied potential vs Ag/AgCl. The Pourbaix diagram shows that CuFeO_2_ is stable over a limited pH-potential window. This Pourbaix diagram is consistent with measurements by Read et al.,^21^ which showed that CuFeO_2_ thin films demonstrated HER in weakly alkaline electrolyte and reported reasonable stability but noted that at acidic pH, the material degrades rapidly. Similarly, Prevot et al.^7^ reported that their sol-gel processed CuFeO_2_ showed water reduction activity when operated under pH 9–10. The experimental photoelectrochemical (PEC) conditions from Read et al. and Prevot et al. both fall within the predicted stability region, hence qualitatively validating the Pourbaix diagram derived by Ferri et al. Using this diagram as a guide, our experimental conditions are marked in a yellow star representing CO_2_-purged at pH 6.9 and +0.22 V vs RHE (−0.4 V vs Ag/AgCl) and a green star representing N_2_-purged at pH 8.9 and +0.33 V vs RHE (−0.4 V vs Ag/AgCl). We see that the surface stability nearly borders the phases of several reduced iron species, and it can be argued that the enhanced dissolution of iron in the CO_2_-purged electrolyte is merely a pH effect. To address whether this is the case, we ran a series of XPS control experiments within this unstable region on the Pourbaix diagram to understand the possible role of CO_2_ beyond simply changing the thermodynamic stability of CuFeO_2_ by altering the solution pH.

**Figure 3:**
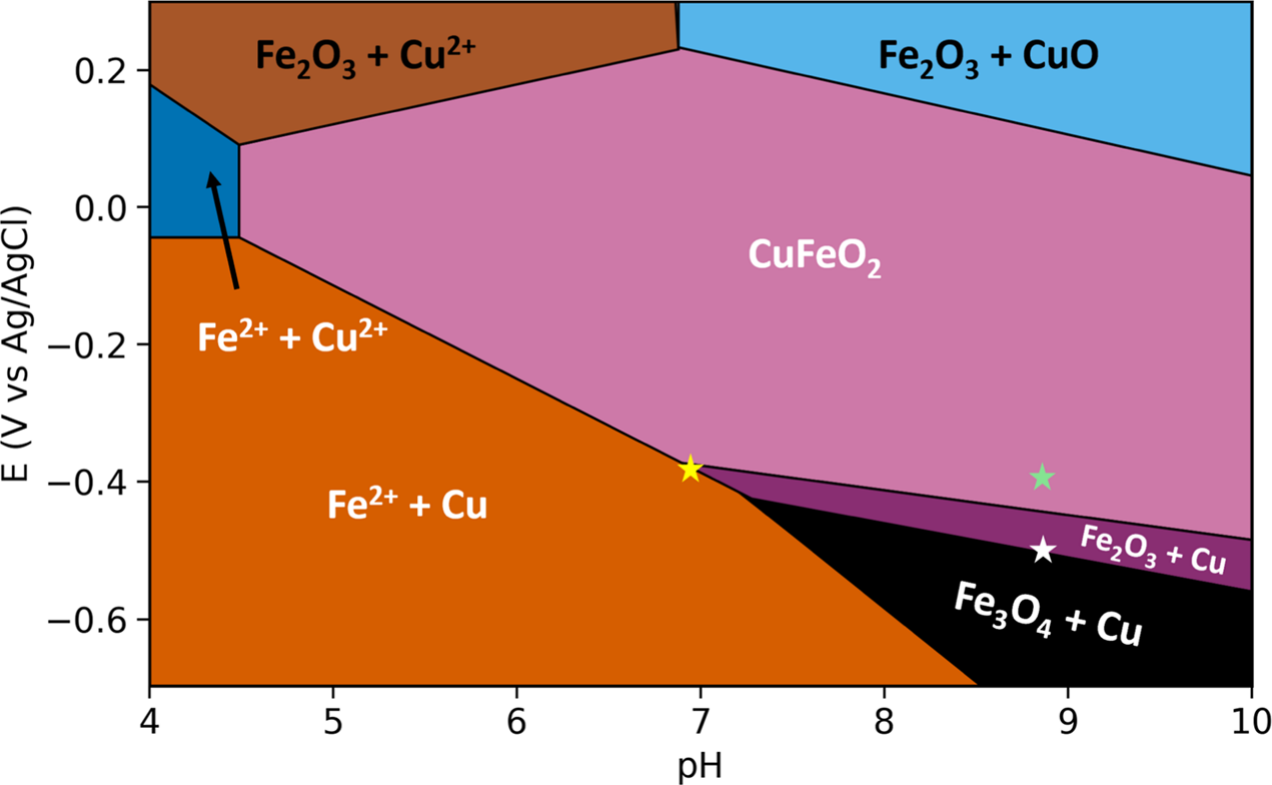
Pourbaix diagram of CuFeO_2_ based on the experimental formation free energy. The yellow and green star represents the CO_2_- and N_2_-purged conditions, respectively. The white star represents the control experimental condition. Adapted from Ferri et al.^26^ Copyright 2019. American Chemical Society.

Figure 4 shows the results of two experiments in which we choose different experimental conditions around this point on the Pourbaix diagram designed to explore the relative stability of CuFeO_2_ as a function of pH and bias near the boundary seen in the Pourbaix diagram between stable CuFeO_2_ and iron leaching. First, we mimic the conditions of CO_2_R (pH and potential), by titrating a N_2_-saturated solution with HCl to match the pH obtained from CO_2_ purging but without the presence of dissolved CO_2_ (Figure 4b). This point is also represented by the same yellow star in the Pourbaix diagram. Here, we find that iron­(III) remains largely stable under these conditions, only losing 33% of the surface iron compared to 100% loss of surface iron under CO_2_-purged conditions, despite identical pH. As a second control, we also change the absolute applied bias from −0.40 V to −0.51 V vs Ag/AgCl while keeping the pH of the N_2_-purged electrolyte constant to match the same applied potential of +0.22 V as the CO_2_-purged electrolyte on the pH-dependent RHE scale. This point is represented by a white star on the Pourbaix diagram, according to which this condition falls within the thermodynamic stable region for iron­(III). However, our results, which can be seen in Figure 4c, show that we lose 78% of the surface iron indicating that the Pourbaix diagram, which describes the bulk thermodynamic stability of the material, may not fully account for interfacial or nonequilibrium conditions. This is consistent with previous first-principles calculations, which demonstrate that accounting for interfacial kinetics requires treatment of nonequilibrium effects and cannot be fully captured using a traditional Pourbaix diagram.^27^ Although the 78% loss of surface iron is not negligible, this is still less than the 100% loss of iron observed under CO_2_-purged conditions, indicating that the thermodynamic influence of CO_2_ saturation on electrolyte pH or RHE potential cannot explain the enhanced currents and greater instability of the CuFeO_2_ catalyst observed under the CO_2_R condition. Additional control experiments were performed (see Supporting Information) in a CO_2_-saturated electrolyte to decouple the roles of illumination and applied bias. Experiments under illumination without applied bias and under applied bias without illumination show that iron leaching occurs under both conditions, where total iron loss is achieved under applied bias even without illumination, while illumination alone also induces significant iron loss. These results demonstrate that catalyst degradation is not solely caused by the electrolyte but arises from a combination of bias and illumination best described as photoelectrochemical corrosion rather than photocorrosion.

**Figure 4:**
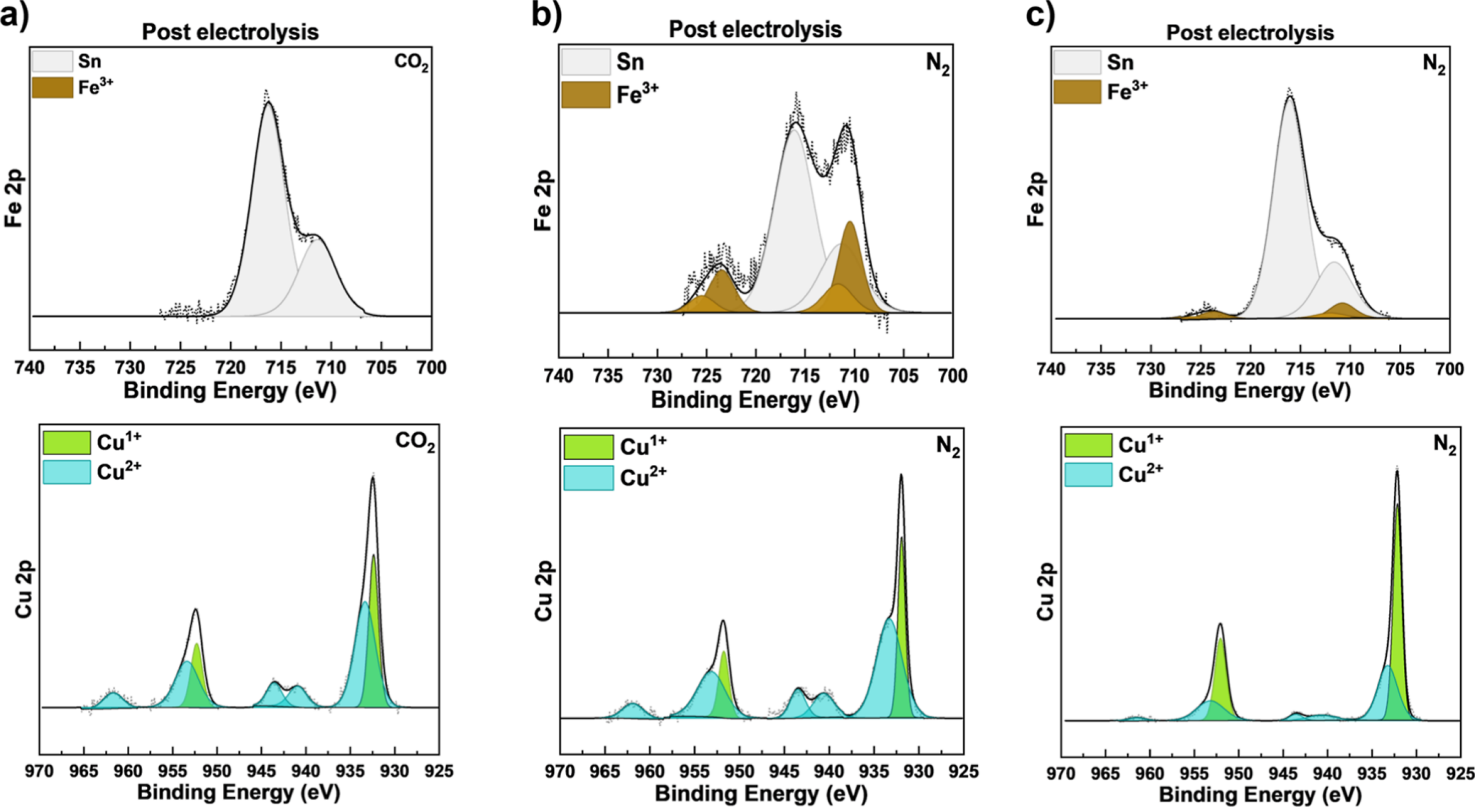
Post-electrolysis XPS of CuFeO_2_ films after applying +0.22 V vs RHE and light illumination in 0.1 M NaHCO_3_ with (a) CO_2_ purging, (b) N_2_ purging and HCl titration, and (c) N_2_ purging.

### Chemical Complexation Pathways Driving Iron Leaching

In Figure 4, we observed some but not total iron loss from the CuFeO_2_ surface under photoelectrochemical conditions in N_2_ saturation, indicating that electrochemical reduction alone is not sufficient to reproduce the total iron depletion, even when accounting for thermodynamic differences predicted in the Pourbaix diagram. To understand how CO_2_ purging can give rise to increased iron leaching, which cannot be explained simply as a change in solution pH or the RHE potential of the electrode, we consider the possible influence of CO_2_ on the interfacial concentration of the species present in the electrolyte. It has been reported that bicarbonate can serve as a proton source for the HER.^28,29^ Due to the higher acidity of bicarbonate (p*K*_a_ = 10.3) compared to that of water (p*K*_a_ = 14), bicarbonate reduction is favored at mild cathodic potentials, while water reduction dominates at more negative cathodic potentials due to mass transport. We note that the direct byproduct of bicarbonate reduction is carbonate, which is known to be a strong chelating agent for iron­(II).

We consider that under CO_2_ purging conditions, it is possible that bicarbonate reduction is favored due to the slight decrease in electrolyte pH, while in N_2_-purged electrolyte, water reduction is more favored. If correct, this would result in a nonequilibrium accumulation of carbonate at the CuFeO_2_ electrode/electrolyte interface in CO_2_-purged electrolyte, where carbonate complexation with iron­(II) generated by photoexcitation of the CuFeO_2_ bandgap would favor reductive dissolution of iron.

To evaluate whether this contributes to the significantly lower stability of CuFeO_2_ in the CO_2_-purged electrolyte than in the N_2_-purged electrolyte, the following two experiments were performed: first, we measured CuFeO_2_ stability in the N_2_-purged 0.1 M NaHCO_3_ electrolyte with the addition of varying concentrations of Na_2_C_2_O_4_ (sodium oxalate) ranging from 0 to 50 mM. Because oxalate is known to strongly chelate iron­(II) (similar to carbonate), evaluating the CuFeO_2_ stability as a function of oxalate concentration reveals whether complexation promotes CuFeO_2_ corrosion via enhanced iron leaching. Second, we performed in situ Raman spectroscopy (SERS) to investigate the speciation of bicarbonate and carbonate near the electrode surface in N_2_- and CO_2_-purged electrolytes as a function of applied potential. This shows whether Faradaic current results in a nonequilibrium accumulation of carbonate near the cathode surface during reaction conditions, which would favor the leaching of iron­(II).

Figure 5a reproduces the electrolysis conditions from Figures 2c and 4a, but instead of CO_2_, Na_2_C_2_O_4_ was added at increasing concentrations. Importantly, Na_2_C_2_O_4_ is a base, which unlike CO_2_, will increase rather than decrease the pH. This indicates that if electrolyte pH governs CuFeO_2_ stability, we should expect reduced leaching of iron, while if interfacial chelation governs CuFeO_2_ stability, we should expect increased leaching of iron. As shown in the Fe 2p XPS scan, we observe a systematic decrease in the iron­(III) peak intensity with increasing oxalate concentration. The quantitative analysis in Figure 5b shows that the iron atomic fraction decreases from 35% in the as-prepared sample to 4% with 50 mM Na_2_C_2_O_4_ present in the electrolyte. These experiments were performed at a fixed absolute potential of −0.40 V vs Ag/AgCl corresponding to +0.33 V vs RHE for the oxalate-free, N_2_-purged electrolyte and were cycled for time periods (7 min) identical with those of the other electrolysis experiments. Note that although the addition of oxalate increases the electrolyte pH, we still observe greater iron leaching with increasing oxalate concentration, confirming that the role of oxalate is not to alter the position on the CuFeO_2_ Pourbaix diagram but rather chelation of iron­(II) by solution phase oxalate-enhanced iron dissolution. These results establish the fact that the total loss of iron under CO_2_ conditions is not solely driven by the pH-dependent CuFeO_2_ Pourbaix diagram but is likely facilitated by a CO_2_-derived chelating species at the interface. The successful replication of iron leaching using a known chelator supports the hypothesis that a CO_2_-derived species promotes iron dissolution through a similar complexation mechanism.

**Figure 5:**
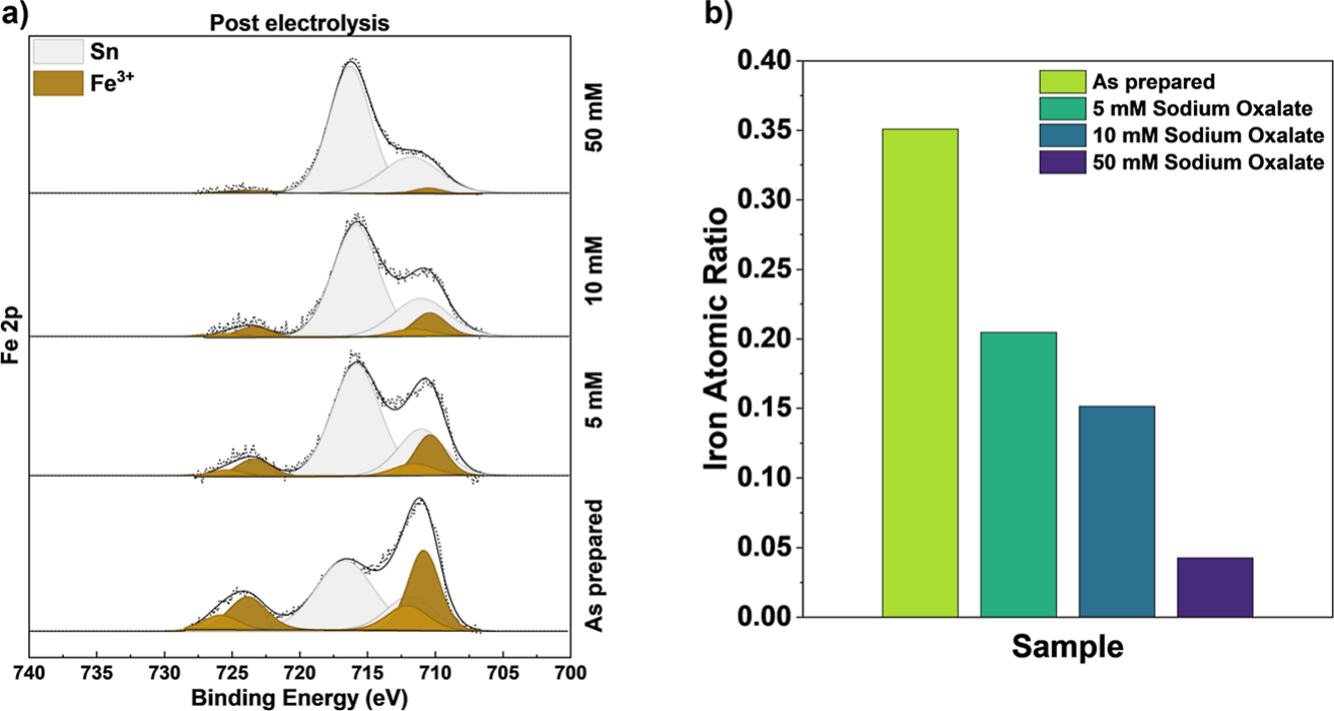
(a) Fe 2p XPS of CuFeO_2_ under N_2_-saturated 0.1 M NaHCO_3_ with 5, 10, and 50 mM sodium oxalate. (b) Quantification of iron atomic ratio with increasing sodium oxalate concentrations.

### Probing Interfacial (Bi)­carbonate Dynamics by In Situ SERS

We now consider the effect of CO_2_ on the speciation of bicarbonate and carbonate at the CuFeO_2_ surface during photoelectrocatalysis. While CO_2_ is purged into the NaHCO_3_ electrolyte, an equilibrium between CO_2(g)_, bicarbonate_(aq)_, and carbonate_(aq)_ is established, and this equilibrium is dependent on the pH of the electrolyte according to the following equations:^30–32^

1 $$CO_{2}+H_{2}O⇌HCO_{3}^{-}+H^{+}$$

2 $$HCO_{3}^{-}⇌CO_{3}^{2-}+H^{+}$$

To measure the bulk and interfacial speciation of bicarbonate and carbonate in N_2_- and CO_2_-purged electrolytes as a function of applied potential, we employed Raman spectroscopy. Because bicarbonate and carbonate are both present in these measurements, we use the term (bi)­carbonate to refer to these species collectively. Raman measurements were conducted both in the bulk electrolyte as a function of pH as well as at the electrode surface as a function of applied potential. For the surface measurements, we employed a bare copper electrode rather than CuFeO_2_. The reason for this is 2-fold: first, in these experiments, we are not probing the surface-specific chemistry of CuFeO_2_; rather we are investigating how the relative concentration of (bi)­carbonate varies in the near-surface region as a function of N_2_ and CO_2_ purging to evaluate whether carbonate can selectively accumulate and contribute to iron dissolution. Second, copper metal is plasmonic, providing a strong SERS signal, resulting in a significantly improved signal-to-noise ratio, which is required for these surface-sensitive measurements. Additional details of the bulk Raman and SERS measurements as well as comparative Raman spectra of CuFeO_2_ deposited on FTO electrode is provided in the Supporting Information. Below, we further show that under CO_2_R conditions, CuFeO_2_ and copper surfaces behave quite similarly due to rapid dissolution of iron, indicating that CuFeO_2_ rapidly evolves to produce copper metal under CO_2_R reaction conditions.

Figure 6a shows the bulk Raman spectra obtained in 1 M NaHCO_3_ while changing the solution pH. The two peaks at 1018 and 1070 cm^–1^ represent the vibrations of bicarbonate and carbonate, respectively. The vibrational mode of bicarbonate is associated with the stretch of C–OH (*A*′′), whereas the vibrational mode of carbonate is associated with the total symmetric C–O stretch (*A*_1_′).^33,34^ Here, the peak area ratio of carbonate:bicarbonate changes as a function of pH, which we can detect with good sensitivity and resolution. Therefore, the carbonate:bicarbonate peak area ratio can be used to monitor changes in the (bi)­carbonate speciation as well as the interfacial pH during reaction.^31,35^

**Figure 6:**
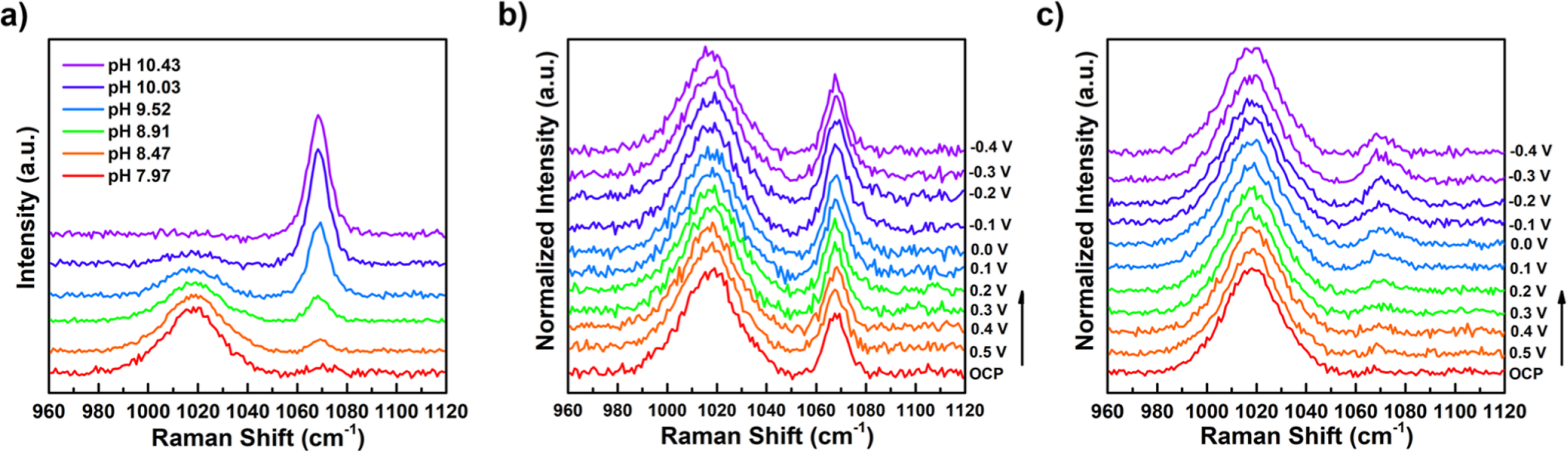
(a) Bulk Raman spectra of copper in different bulk pHs and (b,c) in situ SERS spectra of copper in 1 M NaHCO_3_ with potential sweeping from OCP to −0.4 V under (b) N_2_ purging and (c) CO_2_ purging.

To accomplish this, in situ SERS measurements were performed in 1 M NaHCO_3_ under N_2_- and CO_2_-saturated electrolytes while sweeping the bias to monitor the change of (bi)­carbonate speciation at the interface as a function of potential. Figure 6b,c shows the SERS spectra obtained in the N_2_- and CO_2_-saturated electrolytes, respectively. The potential sweep ranged from open-circuit potential (OCP) at ∼0.5 V to −0.4 V, which corresponds to the potential window where iron dissolution of CuFeO_2_ films was observed during the photoelectrocatalytic reaction under CO_2_ purging. Figure 6b shows that under N_2_ purging, the peak intensity of both the bicarbonate and carbonate remains unchanged as the applied potential is swept to more negative values. The results of the equivalent experiment in CO_2_-purged electrolyte is shown in Figure 6c. In this measurement, the carbonate peak intensity begins lower, as expected for the more acidic, CO_2_-purged electrolyte. However, this feature noticeably increases with applied potential relative to the bicarbonate peak. This is in contrast to the N_2_-purged electrolyte where no change in the carbonate:bicarbonate peak ratio is observed. This can be seen more clearly in Figure 7, which plots the effective pH obtained by the carbonate:bicarbonate peak ratio as a function of applied potential for N_2_- and CO_2_-purged electrolyte. As shown, for the N_2_-purged electrolyte, the pH is constant both in the bulk and at the surface as a function of applied potential. In contrast, in a CO_2_-purged electrolyte, the bulk pH is constant; however, the surface carbonate peak intensity increases with cathodic bias, reflecting an increase in the interfacial pH under CO_2_-purged reaction conditions.

**Figure 7:**
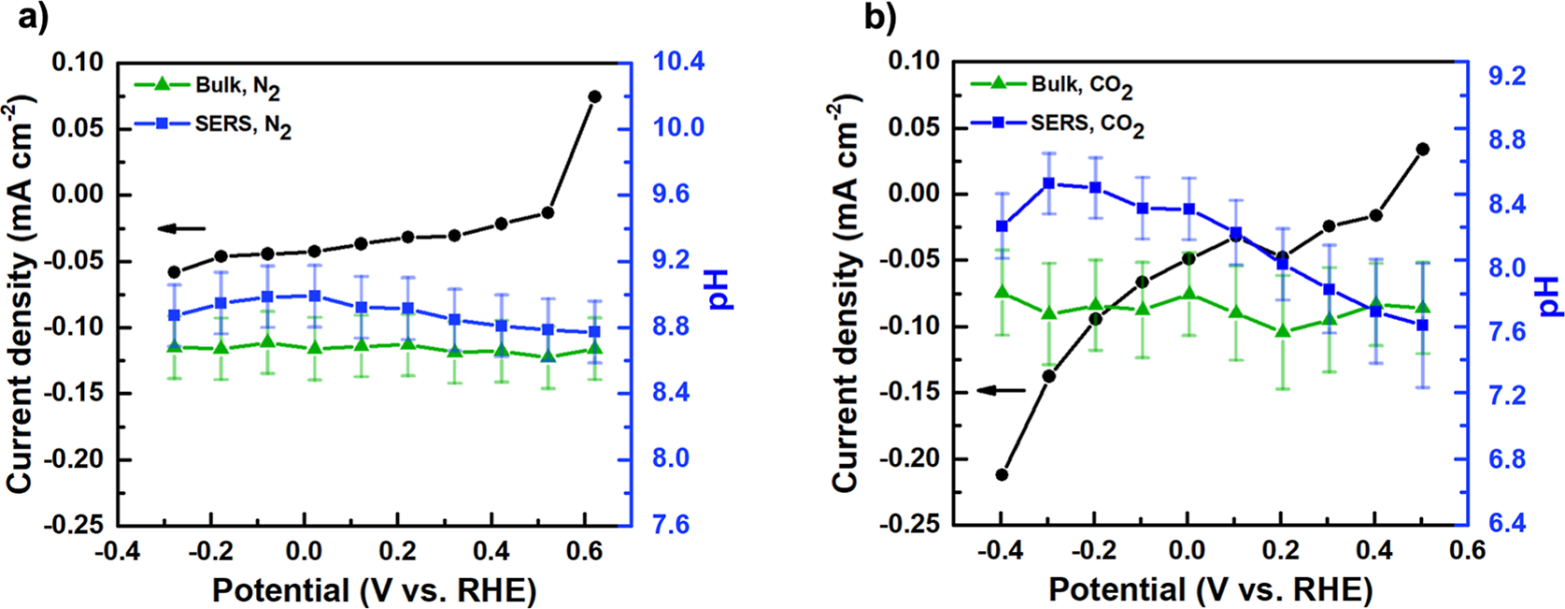
pH and current density as a function of applied bias under (a) N_2_ purging and (b) CO_2_ purging.

As shown in Figure 6c as well as Figure S12 of the Supporting Information, this change in the apparent pH near the electrode surface is a result of increasing carbonate peak intensity, while the bicarbonate peak intensity remains nearly constant. This finding that in the N_2_-purged electrolyte, the surface and bulk pH remain equal, while in the CO_2_-purged electrolyte, the surface pH increases relative to the bulk provides evidence that the CO_2_-saturated electrolyte experiences a nonequilibrium accumulation of carbonate near the electrode surface beginning with the onset of Faradaic current. This is consistent with previous findings that bicarbonate reduction shows an earlier onset potential compared to water reduction.^28^

Note that the HER will increase the local pH regardless of whether bicarbonate or water serves as the primary proton donor. However, because water reduction yields hydroxide as the primary reaction product, in this case, the increase in carbonate concentration proceeds via the equilibration between hydroxide and bicarbonate, which are mediated by sluggish kinetics.^28,36,37^ This will result in a diffuse distribution of carbonate ions with respect to the distance from the electrode surface. In contrast, bicarbonate reduction, which is favored in the CO_2_-purged electrolyte, results in the direct production of carbonate resulting in a high local concentration at the electrode/electrolyte interface. This finding supports the hypothesis that in the CO_2_-purged electrolyte, the nonequilibrium speciation of carbonate near the electrode surface acts to destabilize CuFeO_2_ by promoting iron dissolution.

Finally, we show that owing to the rapid loss of iron from CuFeO_2_ photocathodes in CO_2_-saturated electrolyte, CuFeO_2_ displays a surface chemistry very similar to that of polycrystalline copper metal under conditions relevant for CO_2_R. This can be seen from potential-dependent spectra of CO adsorbed on CuFeO_2_ (Figure 8a) and on polycrystalline copper (Figure 8b). These spectra are measured using vibrational sum frequency generation spectroscopy (VSFG) in CO-purged Na_2_CO_3_ electrolyte, and details of these measurements can be found in the Supporting Information. The purpose of this experiment is to illustrate how the photoelectrochemical corrosion observed above affects the reaction mechanism of CO_2_R on a CuFeO_2_ catalyst. This is demonstrated here by using CO adsorption to the catalyst under reaction conditions, where the CO vibrational frequency serves as a sensitive reporter of catalyst composition and surface structure. Because the vibrational spectrum of adsorbed CO is sensitive to both the composition and the structure of a catalyst surface,^38–42^ this measurement serves as a local probe of the electrode surface chemistry. For example, this can be seen in Figure S15 of the Supporting Information, which shows very different vibrational frequencies for CO adsorbed on copper and silver electrodes. Interestingly, we find that CO adsorption to CuFeO_2_ shows a similar onset potential and nearly identical vibrational frequency as that of the copper metal electrode. This indicates that by the onset of CO adsorption at ∼−0.3 V vs RHE, the CuFeO_2_ surface has been largely converted to copper. Here, we do not comment specifically on the oxidation state of the copper during the reaction. It has been predicted that even at these cathodic potentials, a significant fraction of copper can exist as copper oxide,^40,43–46^ and this is further supported by recent time-resolved VSFG measurements for CO on copper electrodes.^40^ Consistent with the accumulation of carbonate near the electrode surface in the CO_2_-saturated electrolyte, it is possible that oxidation of the copper surface is facilitated by specific adsorption of carbonate ions,^47,48^ which would favor a malachite-like surface phase.^44,49^ Regardless of the exact oxidation state or surface phase of copper catalysts during CO_2_R, these VSFG measurements confirm that CuFeO_2_ behaves as Cu metal under reaction conditions, further supporting the conclusion that CuFeO_2_ is prone to rapid dissolution of surface iron and that this dissolution is even more severe under CO_2_R reaction conditions compared to HER reaction conditions.

**Figure 8:**
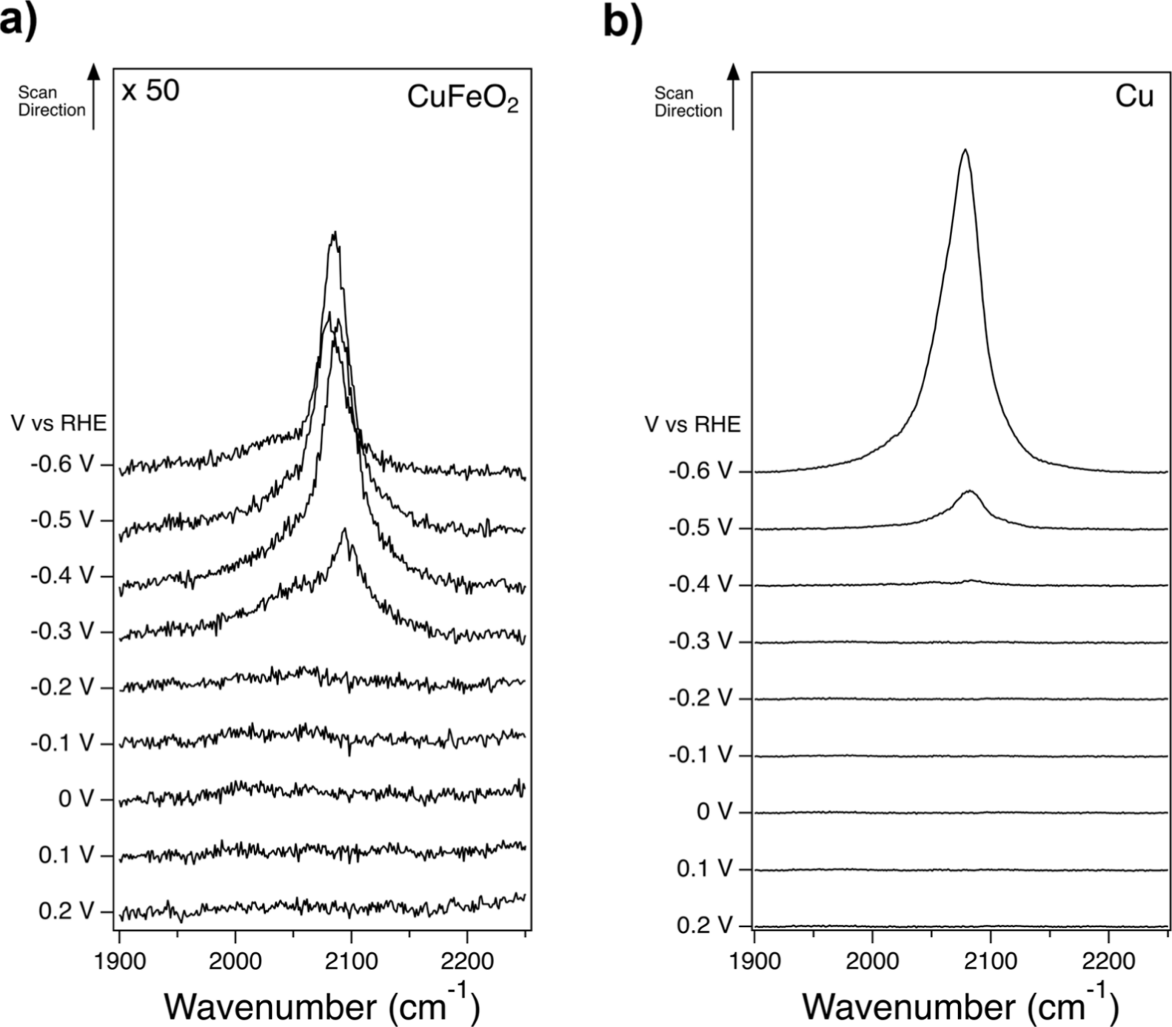
VSFG spectra taken in CO-saturated 0.05 M Na_2_CO_3_ showing the potential-dependent CO adsorption to (a) CuFeO_2_ and (b) metallic copper.

## Conclusions

Through a combination of photoelectrochemical and spectroscopic analyses, we have investigated the mechanistic origin of CuFeO_2_ instability under CO_2_R conditions. Despite its promising optoelectronic properties, CuFeO_2_ is not an effective CO_2_R photocatalyst. The photoelectrochemical measurements revealed a 2-fold increase in photocurrent under the CO_2_-saturated electrolyte compared to N_2_, yet gas chromatography and NMR analysis failed to show any products resulting from CO_2_R, indicating that the enhanced current does not arise from the photocatalytic activity of the material. Ex situ XPS measurements established that CuFeO_2_ undergoes a total loss of iron under the CO_2_R conditions, in contrast to its comparatively higher stability under HER conditions. Control experiments demonstrate that this instability cannot be fully explained by pH or potential dependent thermodynamic limits but originates from nonequilibrium speciation at the interface of the catalyst. In situ SERS measurements further revealed that under CO_2_ purging, cathodic bias leads to a nonequilibrium increase of carbonate at the catalyst interface, which binds with iron and facilitates the rapid conversion of CuFeO_2_ to copper. Together, these results establish that CuFeO_2_ instability under CO_2_R conditions arises from nonequilibrium interfacial chemistry that drives the reductive dissolution of iron. More broadly, this work highlights that photocathode stability under CO_2_R is not solely determined by thermodynamic stability but also critically governed by dynamic interfacial speciation under reaction conditions. These insights provide a mechanistic foundation for designing earth-abundant oxide photocatalysts that can maintain their structural stability under CO_2_R conditions.

## Supplementary Material



## Notes

Materials; CuFeO_2_ synthesis; thin film preparation; photoelectrochemical measurements; product detection and quantification; XPS measurements; in situ SERS measurements; and in situ VSFG Measurements (PDF)
