# Supplementary material for: Carbonate-Enhanced Photoelectrochemical Corrosion Limits the CO2 Reduction Reactivity on CuFeO2 Delafossite Photocathodes
Source: J Phys Chem C Nanomater Interfaces. 2026 Mar 26;130(14):5258–67. doi: 10.1021/acs.jpcc.5c08145 (PMC13071908; doi:10.1021/acs.jpcc.5c08145)
Supplement: Supplementary file 1 [file jp5c08145_si_001.pdf]

Supporting Information: Carbonate-Enhanced Photoelectrochemical Corrosion Limits the CO_2 Reduction Reactivity on $CuFeO_2$ Delafossite Photocathodes

Piyush Anil Kumar Sharma,[†] *Jaclyn A. Rebstock,*[†] *Ting-Rong Ko, Emma Pollock, Jeffersson Feutseu, Julia C. Lam, Patrick M. Woodward, and L. Robert Baker**

Department of Chemistry and Biochemistry, The Ohio State University, Columbus, Ohio 43210, United States

[†]These authors contributed equally to this work.

[*]Corresponding author

Email: baker.2364@osu.edu

Phone: +1 (614)- 292- 2088

Contents

S1. Materials 2
S2. $CuFeO_2$ Synthesis 2
S3. Thin Film Preparation 3
S4. Photoelectrochemical Measurements 6
S5. Product Detection and Quantification 7
S6. XPS Measurements 8
S7. In situ SERS Measurements 13
S8. In situ VSFG Measurements 17
References 21

S1. Materials

2.2 mm thick FTO glass was purchased from Sigma Aldrich. Cu_2O (99.99%) and Fe_2O_3 (99.99%) powders for the $CuFeO_2$ synthesis were purchased from Sigma Aldrich. For photoelectrochemical experiments, sodium bicarbonate ($NaHCO_3$) electrolyte solutions were prepared using sodium bicarbonate (99.7%) and milli-Q water. For product detection and VSFG experiments, high-purity Na_2CO_3 (99.999%) were used instead. The Ag/AgCl (3 M NaCl) aqueous reference electrode was purchased from BASi. Carbon dioxide (CO_2) (99.999%), nitrogen (N_2) (99.999%), and CO (99.999%) were purchased from Praxair.

S2. $CuFeO_2$ Synthesis

$CuFeO_2$ was prepared using a conventional solid-state method.[1] Stoichiometric amounts of Cu_2O and Fe_2O_3 were mixed and calcinated at 1000 °C for 14 hours under Ar flux. The calcinated mixtures were then finely grounded, pelletized, and sintered at 1100 °C for 24 hours. The samples were characterized using powder X-ray diffraction (XRD). Figure S1a and b show the XRD and SEM of the $CuFeO_2$ powder. Rietveld refinement of the powder XRD shows that $CuFeO_2$ is in space group R-3m (Rwp = 2.27%). The difference curve is shown in blue. Black tick marks correspond to reflections from the 3R polymorph of $CuFeO_2$.

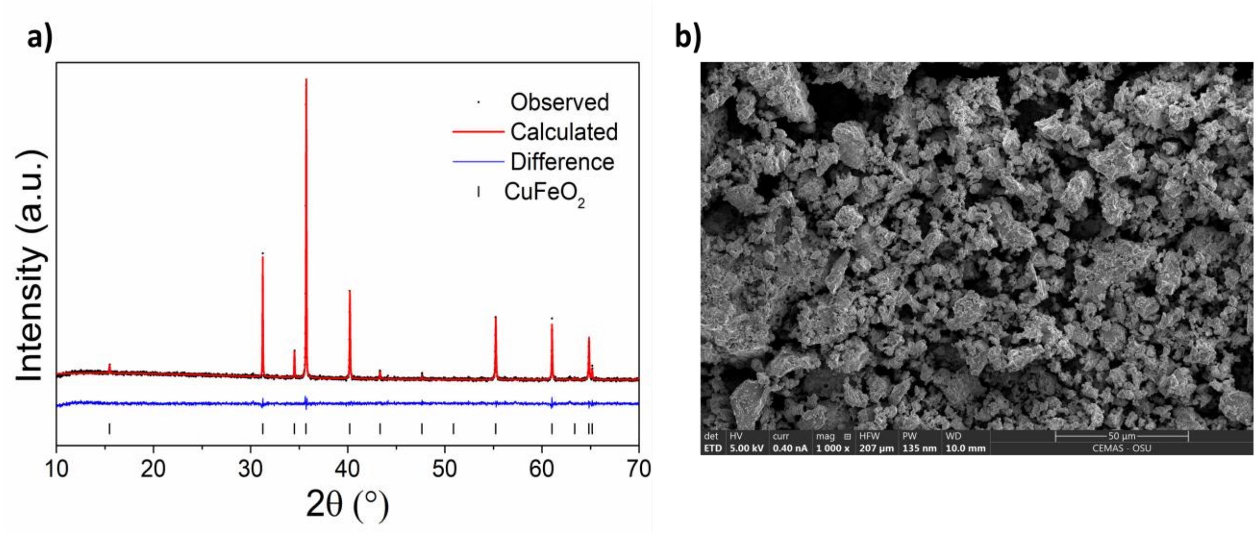

Figure S1. (a) XRD and (b) SEM of the prepared $CuFeO_2$ powder.

S3. Thin Film Preparation

Fluorine-doped tin oxide (FTO) substrates were cut into 1.5 cm^2 pieces and were cleaned with basic piranha solution (5 parts water to 1 part hydrogen peroxide to 1 part ammonium hydroxide) for 20 min. After piranha cleaning, the substrates were rinsed with milli-Q water and dried with N_2. A $CuFeO_2$ ink slurry was made by mixing the synthesized $CuFeO_2$ with iso-propyl alcohol and Nafion (5 wt%). The precursor solution was sonicated for 20 min before drop casting on the FTO substrate over a hot plate at 100 °C. We drop cast a volume of 150 μL of the precursor to produce a final weight of 3.6 mg of $CuFeO_2$ on the FTO substrate.

Annealing of the films is required to remove excess Nafion from the drop casted ink slurry precursor. Annealing the films in N_2 vs air at 550 °C for 1 hour showed drastically different photoelectrochemical activity (Figure S2). The N_2-annealed samples showed low dark current, only mildly more active than the bare FTO substrate. However, with air annealing, the

electrochemical dark current and the photoresponse to a chopped light source at 0.5 Hz become significant.

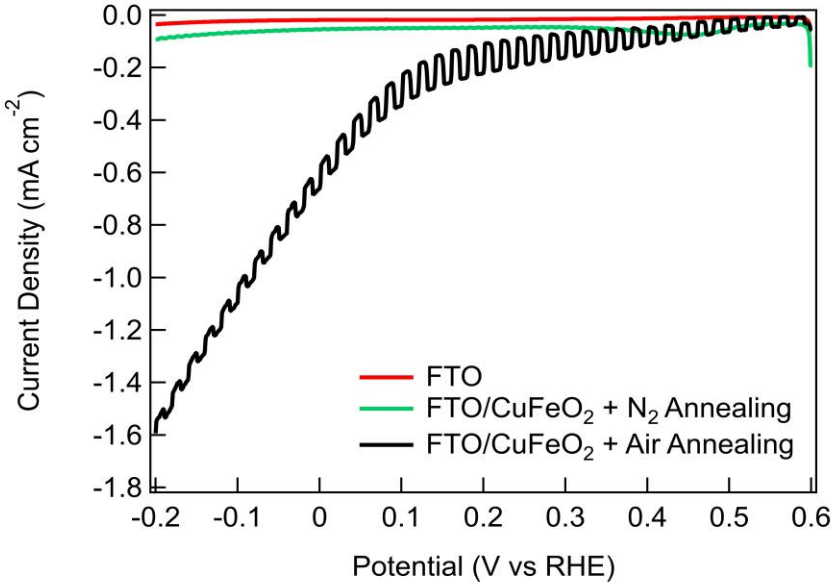

Figure S2. LSVs of $CuFeO_2$ films under N_2 (green) and air-annealing (black) compared to bare FTO substrates (red).

Raman characterization showed that air annealing introduced copper(II) surface species. Figure S3 shows the presence of CuO character which comes from annealing the electrode in an oxygen rich environment (air). Among the 12 vibrational modes within $CuFeO_2$ delafossite, two are Raman-active with E_g and A_{1g} symmetry.[2] Both modes are present (E_g and A_{1g}) at 350 cm^{-1} and 690 cm^{-1}, respectively in the N_2 annealed $CuFeO_2$ film. For reference, a CuO film was tested and shows three Raman-active modes that match well with reported literature values (288, 330, and 621 cm^{-1} with A_g, A_g, and B_g symmetry, respectively).[3] $CuFeO_2$ electrodes in the main manuscript were prepared by annealing in air. These samples distinctly show the $CuFeO_2$ spectral features seen in the N_2 annealed electrodes. However, the presence of CuO's B_g mode at 621 cm^{-1} begins

to appear in the baseline. This indicates that a small fraction of the sample is comprised of CuO defects which lead to the enhanced activity of $CuFeO_2$ as seen in Figure S2.

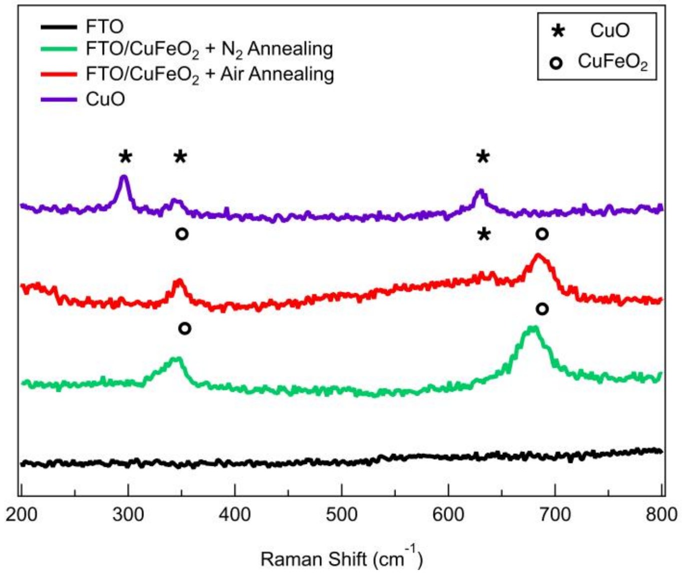

Figure S3. Raman spectra showing effects of air annealing the $CuFeO_2$ thin films.

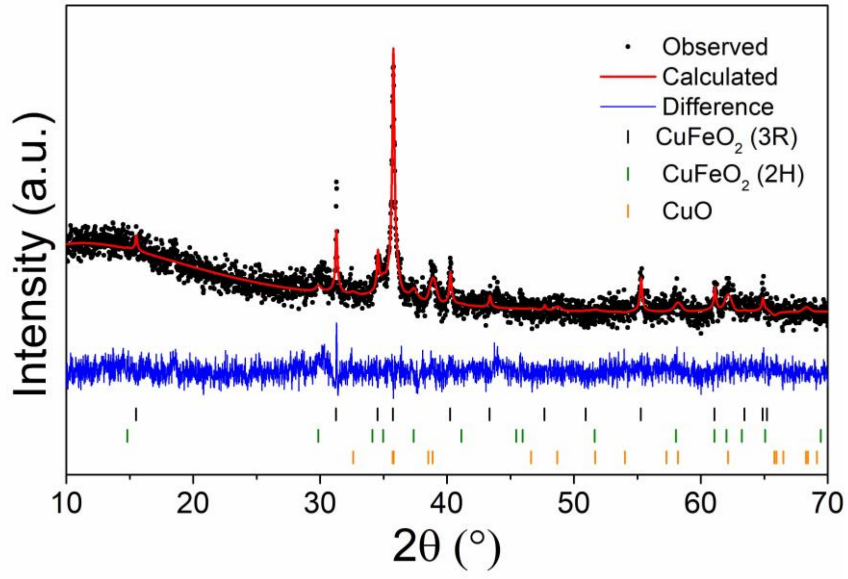

Figure S4. XRD of the $CuFeO_2$ powder annealed at 550 °C.

In Figure S4 the XRD of the annealed powder where we see a minor contribution from CuO (8%). Rietveld refinement of the powder XRD shows that majority of $CuFeO_2$ is present in the 3R

phase (85%) along with a small contribution from the 2H phase (7%). The difference curve is shown in blue. Black and green tick marks correspond to reflections from the 3R and 2H polymorph of $CuFeO_2$ respectively, while the yellow tick mark correspond to reflections from CuO.

S4. Photoelectrochemical Measurements

A Biologic SP-50 potentiostat was used for all electrochemical measurements. A white LED light was used as a light source (Thorlabs, MWWHL3). The output of the lamp was focused onto the sample with a power density of 100 mW/cm^2. Figure S5 shows the spectral profile of the white light source.

Linear sweep voltammetry (LSV) was performed in a three-electrode cell containing a quartz window for light illumination. A Ag/AgCl (3 M NaCl) electrode was used as the reference and a Pt electrode was used as the counter electrode. For LSVs, the potential was swept from +0.6 V to -0.4 V with a sweep rate of 10 mV/s as the light was modulated on and off with a frequency of 0.5 Hz. For XPS and product detection experiments, a constant illumination was used. The catalyst was illuminated by the $CuFeO_2$ side of the sample in all measurements. All applied electrochemical potentials are converted to the reversible hydrogen electrode (RHE) scale using the Nernst equation as follows,

$$E_{RHE} = E_{Ag/AgCl} + 0.209\,V + (0.059\times\mathrm{pH}) \quad (1)$$

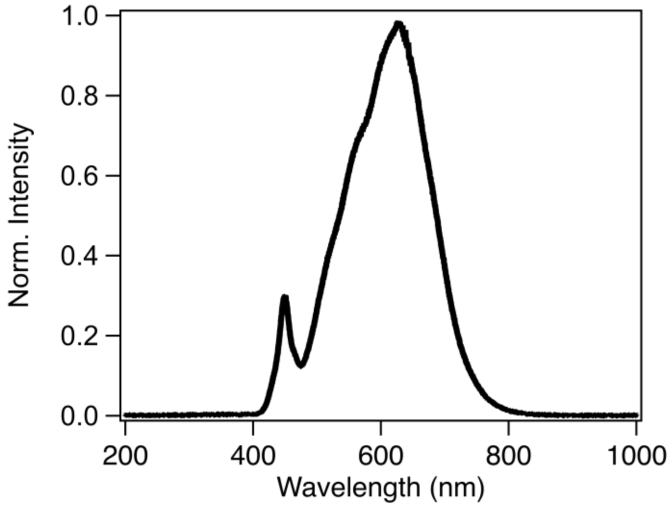

Figure S5. Spectral profile of light source used for LSV and PEC measurements.

S5. Product Detection and Quantification

Gas phase product detection was performed using an electrochemical H-cell in an identical sample and electrode geometry as described above for the photoelectrochemical measurements.

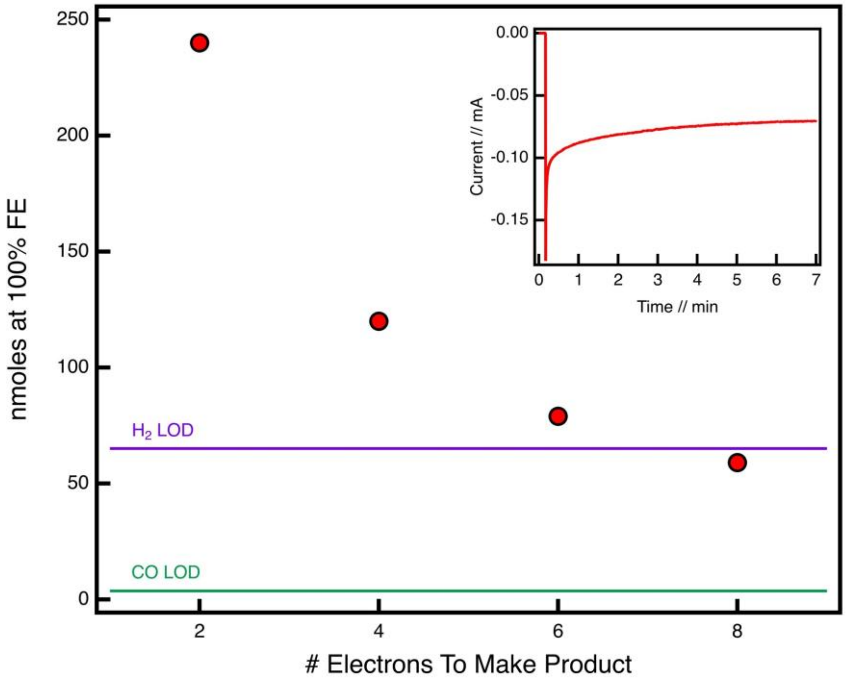

Figure S6. (Inset) Current density measured during fixed electrolysis at +0.22 V with light illumination. (Main Figure) The theoretical product yield based on the measured current density and calculated GC LODs.

The cell head space was sampled using gas chromatography (Agilent 7890B) equipped with a flame ionization detector (FID) and thermal conductivity detector (TCD) for gas products like H_2 or CO, respectively. Either N_2 or CO_2 is purged into the electrochemical cell for 20 min before starting the electrolysis. A blank GC injection is run at the start of the electrolysis, then each GC injection takes 7 min to run through the column and detect products.

Figure S6 (inset) shows the current passed over this first 7 min at a fixed potential (+0.22 V in N_2 purging). After 7 min, the head space was sampled again. No gas phase products were detected under these conditions. Instead, if all of the measured current density seen in the inset of Figure S7 went to products, we calculate how many nmoles of products could be formed, given the amount of electrons it takes to make that product (2 electrons for H_2 or CO). This can be seen as the red dots in Figure S6. The GC LODs for these two products are also calculated by injecting the GC with blanks and evaluating the standard deviation of the baseline. These are also marked on Figure S6. This shows that if the current density measured in 7 min was due to H_2 or CO product detection, we would be able to detect it via GC.

S6. XPS Measurements

XPS was ran on a ThermoFisher Nexsa G2. Following the photoelectrochemical measurements, the $CuFeO_2$ films were transferred for the ex situ XPS analysis without any additional rinsing or washing to avoid any loss of catalyst. Atomic fractions were determined by peak area fittings followed by normalization to relative sensitivity factors (RSF) using the Nexsa G2 RSF library. Spectral fitting was performed with CasaXPS using Gaussian-Lorentzian (GL 30) profiles with a consistent Shirley baseline. The binding energy scale was calibrated using the C 1s peak (BE = 284.8 eV).

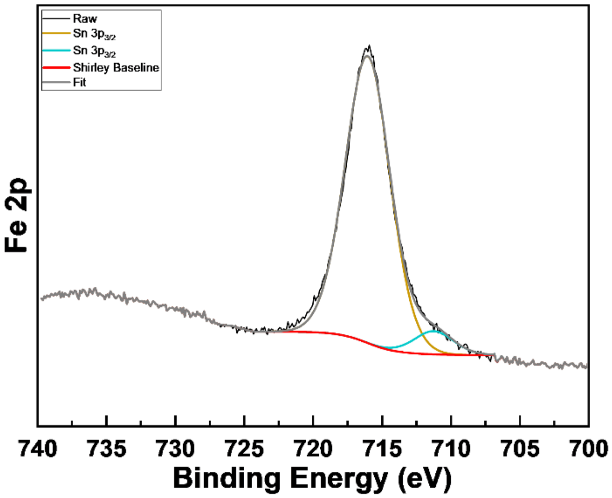

Figure S7. XPS spectrum of FTO substrate.

$CuFeO_2$ Film Transparency: The transparent nature of the film means that the underlying Sn signal from the FTO substrate is visible through the sample and detected during XPS analysis. Figure S7 shows the Fe 2p XPS spectrum of a bare FTO substrate A prominent peak at 715.99 eV can be attributed to the Sn $3p_{3/2}$ orbital of the Sn^{4+} substrate which can be ascribed to SnO_2. [4,5] In addition, a weaker contribution center near 711.19 eV is observed. Because the Sn 3p signal is broad and arises from Sn species present in different chemical environments within the FTO substrate, the overall substrate signal cannot be adequately fit using a single component. Therefore, two constrained Sn $3p_{3/2}$ peaks are used to accurately fit the region with binding energies 715.99 and 711.19 eV and FWHM of 3.78 and 3.70. These Sn fitting parameters are used to deconvolute the Sn $3p_{3/2}$ signal from the Fe $2p_{3/2}$ and $2p_{1/2}$ signal.

Component Fitting: Cu 2p spectra were fit with contributions from Cu^{2+} and Cu^{1+} surface sites. Considering the $2p_{3/2}$ and $2p_{1/2}$ orbitals for each oxidation state, there are four main spectral components in the 2p region. Indicative of the presence of Cu^{2+} sites, satellite features appear, adding three more spectral components to the Cu 2p fitting. The Fe 2p spectral region is convoluted with the underlying Sn oxide from the FTO substrate. The remaining signal is fit to the $2p_{3/2}$ and $2p_{1/2}$ orbitals from Fe^{3+}. Due to the strong spin-orbit coupling of the Fe 2p orbitals,[6] this produces a total of four Fe 2p components. Figure S8 and S9 show the non-background subtracted spectra with their component fittings. The peak position, FWHM and Areas are reported in Table S1.

Component	Binding Energy (eV)	FWHM	Area
Fe^{3+} $2p_{3/2}$ (peak 1)	710.59 ± 0.27	2.92 ± 0.26	--
Fe^{3+} $2p_{3/2}$ (peak 2)	711.86 ± 0.41	3.49 ± 0.05	--
Fe^{3+} $2p_{1/2}$ (peak 1)	723.49 ± 0.26	3.28 ± 0.34	0.5× Fe^{3+} $2p_{3/2}$ (peak 1)
Fe^{3+} $2p_{1/2}$ (peak 2)	725.59 ± 0.27	3.13 ± 0.20	0.5× Fe^{3+} $2p_{3/2}$ (peak 2)
Sn^{4+} $2p_{3/2}$ (peak 1)	711.42 ± 0.30	4.15 ± 0.29	0.4× Sn^{4+} $2p_{3/2}$ (peak 2)
Sn^{4+} $2p_{3/2}$ (peak 2)	716.23 ± 0.29	4.13 ± 0.53	--
Cu^{2+} $2p_{3/2}$	933.47 ± 0.25	2.97 ± 0.27	--
Cu^{2+} $2p_{3/2}$ satellite (peak 1)	940.86 ± 0.37	2.75 ± 0.28	--
Cu^{2+} $2p_{3/2}$ satellite (peak 2)	943.52 ± 0.26	1.86 ± 0.20	--
Cu^{2+} $2p_{1/2}$	953.36 ± 0.26	3.41 ± 0.19	0.5× Cu^{2+} $2p_{3/2}$
Cu^{2+} $2p_{1/2}$ satellite (peak 3)	961.70 ± 0.31	2.51 ± 0.16	--
Cu^{1+} $2p_{3/2}$	932.20 ± 0.18	1.23 ± 0.39	--
Cu^{1+} $2p_{1/2}$	952.05 ± 0.19	1.56 ± 0.41	0.5× Cu^{1+} $2p_{3/2}$

Table S1. Fitting parameters of the XPS components used to fit all the Fe and Cu spectra.

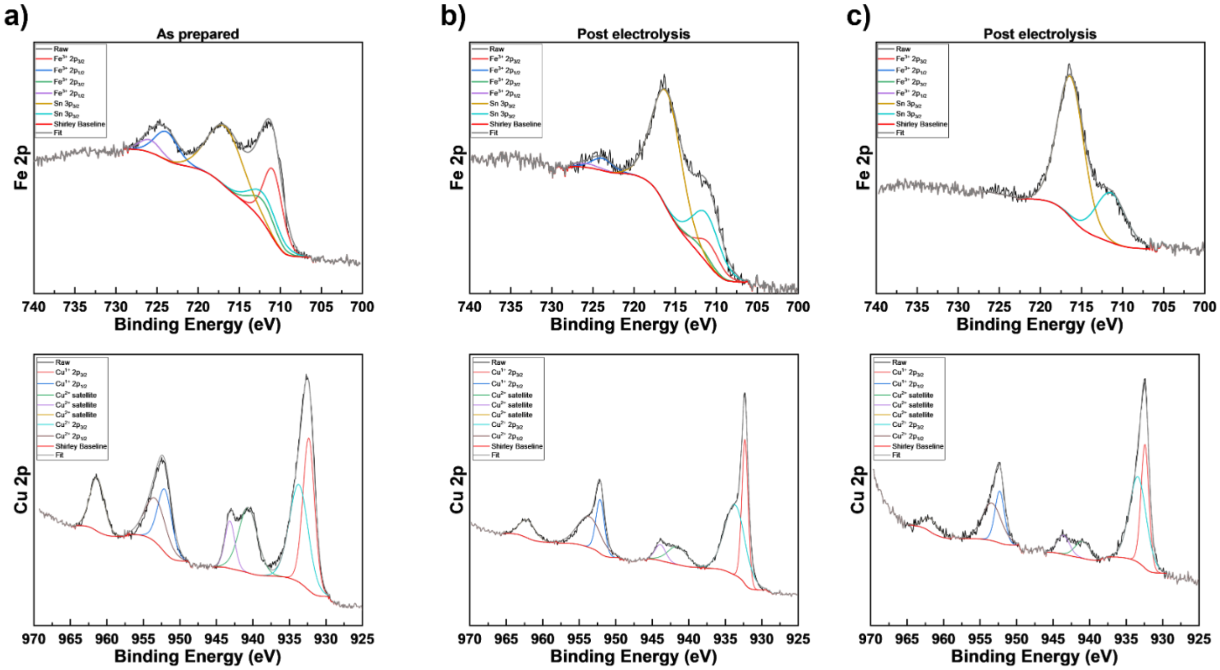

Figure S8. Raw XPS data with Shirley baseline and component fitting of Fe 2p (top) and Cu 2p (bottom) spectra for the $CuFeO_2$ films (a) as prepared, (b) post electrolysis with N_2 purging (c) post electrolysis with CO_2 purging. Baseline-subtracted spectra are given in Figure 1 of the main manuscript.

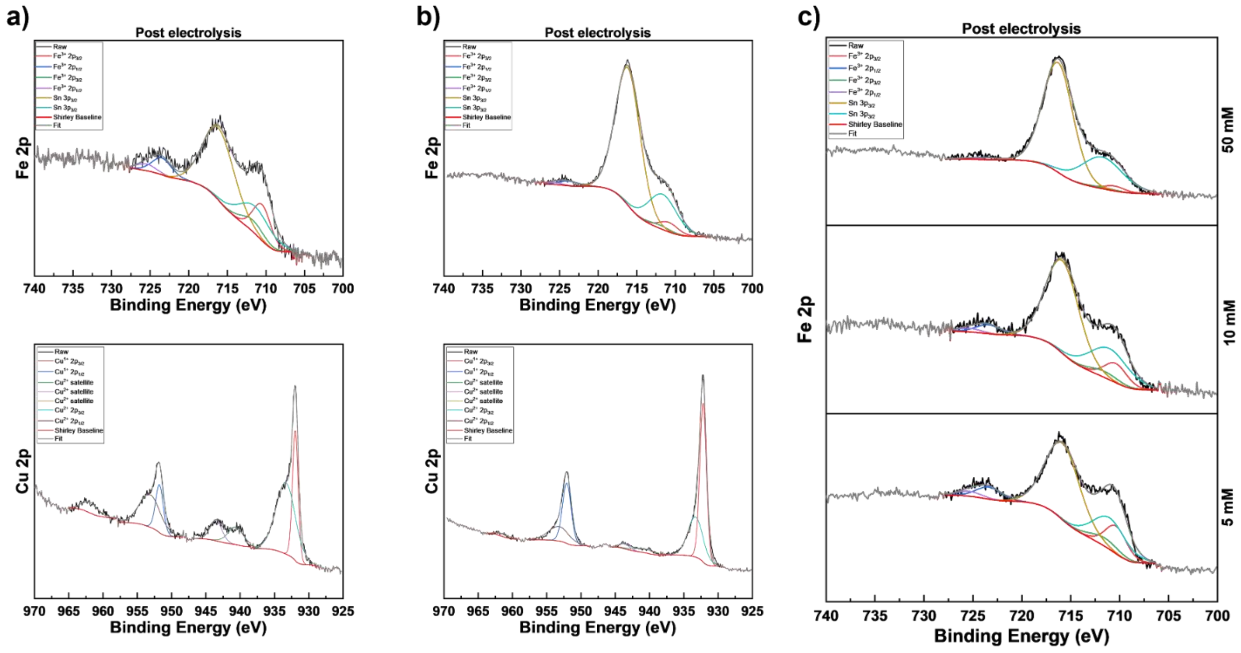

Figure S9. Raw XPS data with Shirley baseline and component fitting of Fe 2p and Cu 2p spectra for (a) N_2 purging with HCl titration, (b) N_2 with a bias overpotential post electrolysis, and (c) oxalate chelation.

Elemental Film Quantification: To quantify the film composition, we monitored the relative surface composition of Fe and Cu using XPS. To avoid artifacts arising from substrates, only spectral components attributed to Fe and Cu originating from the $CuFeO_2$ films are included in the quantification. The Fe atomic ratio reported in the main manuscript is calculated from the fitted peak areas. Specifically, the integrated areas of all Fe 2p components assigned to iron species are summed together after removal of underlying Sn contributions. Likewise, the integrated areas of all Cu 2p components are summed to obtain the total Cu contribution.

The Fe atomic ratio reported in the main manuscript is calculated from the fitted peak areas as follows:

$$Fe\ Atomic\ Ratio = \frac{\frac{A_{Fe^{3+}}}{SF_{Fe\ 2p}}}{\frac{A_{Fe^{3+}}}{SF_{Fe\ 2p}} + \frac{A_{Cu^{2+}}}{SF_{Cu\ 2p}} + \frac{A_{Cu^{+}}}{SF_{Cu\ 2p}}} \quad (2)$$

where A represents the fitted peak areas and SF represents the atomic sensitivity factors of the corresponding features.

Mechanism Controls: To investigate the effect of both the light illumination and the applied bias on the Fe leaching process, we performed three control experiments under CO_2 purging that include no light illumination and no bias (Figure S10a), light illumination with no applied bias (Figure S10b), and no light illumination with an applied bias (+0.22 V vs RHE, Figure S10c). These controls were collected under the same electrolysis conditions given Figures 2c and 4a of the main manuscript where the Fe leaching occurred. In the case of no light and no bias, these results mimic the as-prepared $CuFeO_2$ films pre-electrolysis shown in the main manuscript and show no evidence of Fe leaching, proving that the film instability is not due to the electrolyte itself.

The Fe atomic ratio is 39% compared to 35% measured for the as-prepared film; this small difference arises from the sample-to-sample variation of the film preparation. Light illumination alone causes significant Fe leaching where the Fe atomic ratio drops to 6%. The total loss of Fe can only be replicated with bias.

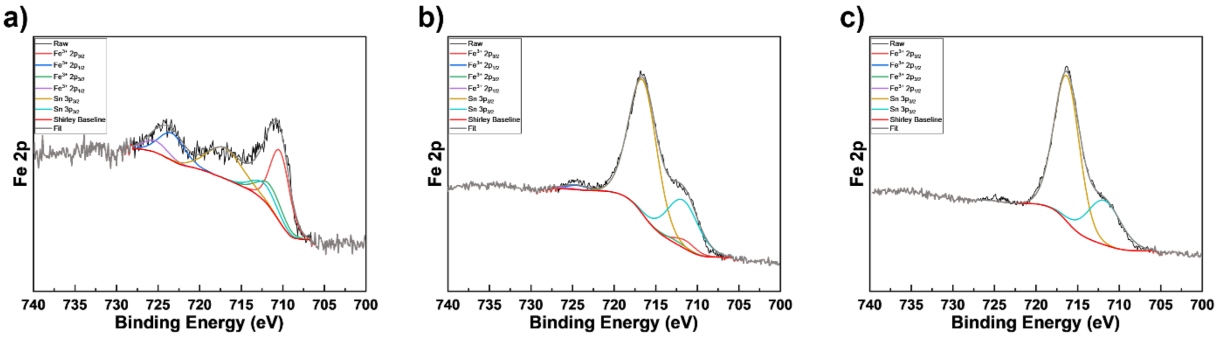

Figure S10. Raw XPS data with Shirley baseline and component fitting of Fe 2p spectra after CO_2 purging and (a) no light and no applied bias, (b) light illumination with no applied bias, and (c) no light with an applied bias (+0.22 V vs RHE).

S7. In situ SERS Measurements

Surface Enhanced Raman Spectroscopy (SERS) was performed using a Renishaw inVia Raman microscope with 785 nm illumination on polycrystalline copper thin films in a home-built electrochemical cell. The thin film polycrystalline copper metal was prepared via electron beam evaporation (Denton DV-502A E-Gun Evaporator) to create a 150 nm copper film. The films were deposited on glass microscope slides that were cleaned with piranha solution and then dried with N_2.

A reservoir containing the electrolyte under purging and a peristaltic pump was used to pump fresh solution into the cell at low flow (1 mL/min) through the cell to avoid fluctuations in the Raman spectra. Prior to the in situ measurements, the copper surface is prepared with three CV cycles to ensure the sample surface was clean. Each potential step was cycled the same so that after the potential step, the current was given 30 s to stabilize before collecting a spectrum. For a

30 s integration time, it takes 2 min to acquire before switching to the next potential. All potentials were collected within the same experiment. As shown in Figure 6a of the main text, the intensity of carbonate and bicarbonate peaks change according to the bulk pH. The integrated Raman peak areas of carbonate and bicarbonate were used for the calculation of relative peak ratio of carbonate:bicarbonate.

A linear relationship can be established between the logarithm of Raman peak area ratio of carbonate:bicarbonate and the bulk pH. This indicates that the integrated Raman peak area can be treated as the concentration of carbonate and bicarbonate.[7] To calculate the interfacial pH or bulk pH, the Henderson-Hasselbach equation is applied.

$$pH = \left[pKa + \log\left(\frac{CO_3^{2-}}{HCO_3^-}\right)\right] + C \tag{3}$$

where C is the average deviation between the pH obtained from carbonate:bicarbonate Raman peak area ratio from bulk Raman spectra in different electrolyte pH (i.e., pH_{Raman}) and the pH measured by the calibrated pH meter (i.e., pH_{meter}).

$$pH_{Raman} = \left[pKa + \log\left(\frac{CO_3^{2-}}{HCO_3^-}\right)\right] \tag{4}$$

$$C = pH_{meter} - pH_{Raman} \tag{5}$$

The pH uncertainty is calculated as the square root of the sum of the square errors from pH_{Raman} and the standard deviation of C, where the pH_{Raman} error depends on the propagated uncertainty in the fitted Raman peak area ratio.

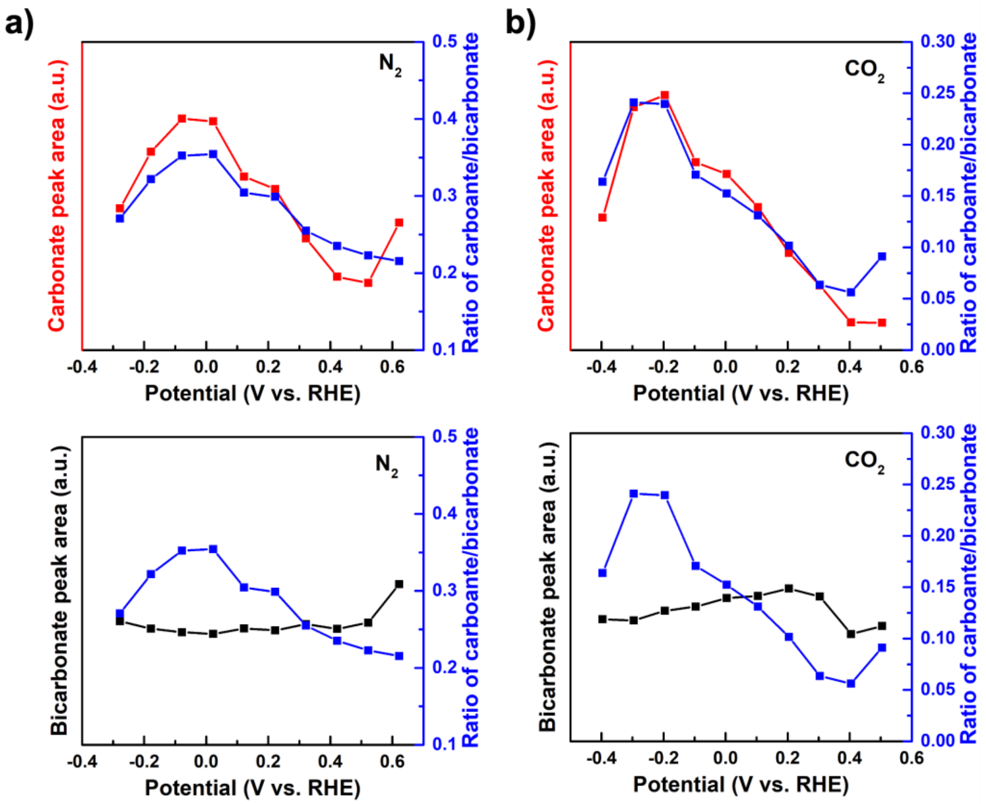

Figure S11. Relations between the carbonate/bicarbonate peak area ratio and the absolute peak areas (a) under N_2 purged and (b) under CO_2 purged electrolyte.

Figure S11 is obtained from the integrated peak area of in situ SERS measurement in 1 M $NaHCO_3$ on copper electrode. The absolute peak area of carbonate is increasing as the ratio of carbonate:bicarbonate arises, while the peak area of bicarbonate remains constant for both N_2- and CO_2-purged electrolyte. This indicates that at the electrode surface, carbonate species is produced as the applied potential is swept to more cathodic. In CO_2-purged electrolyte, the ratio of carbonate:bicarbonate increased by 5-fold from +0.5 V to 0.0 V, while the ratio increased only 1.6 times in N_2-purged electrolyte. This significant difference indicates that purged CO_2 facilitates the production of carbonate at the interface.

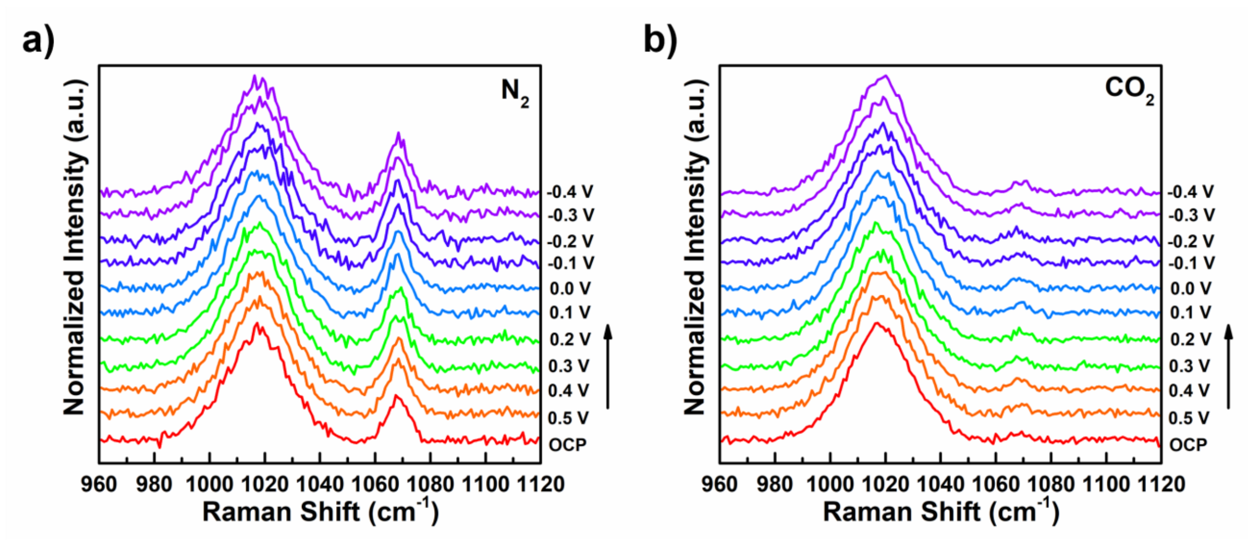

Figure S12. In situ Raman spectra measured in bulk solution with 1 M $NaHCO_3$. The potential is swept from open-circuit potential (OCP) to -0.4 V under (a) N_2 purging and (b) CO_2 purging.

Figure S12(a-b) shows the potential dependent Raman spectra measured from bulk in N_2- and CO_2-purged electrolyte, respectively. As the potential is swept more cathodic, we did not observe any intensity changes in the carbonate peak for both cases. This suggests that the production of carbonate is purely an interfacial effect under the presence of CO_2.

Figure S13 (a-d) presents the in situ Raman spectra recorded at the interface in 1M $NaHCO_3$ with CO_2 purging using (a-b) metallic Cu and (c-d) $CuFeO_2$ deposited on an FTO substrate. Measurements of $CuFeO_2$ deposited on an FTO substrate minimize the interfacial sensitivity due to lack of surface plasmon enhancement making reliable extraction of interfacial pH gradients challenging. This can be seen in Figure S13, which shows large error bars resulting from reduced signal-to-noise and decreased surface sensitivity in the absence of plasmonic signal enhancement. By comparison, the Cu electrode provides a plasmonic enhancement, which increases the interfacial sensitivity to the (bi)carbonate signals, and this enables us to observe the formation of

interfacial pH gradients under applied bias. Thus, the metallic Cu substrate was used primarily to enable plasmon enhanced Raman measurement of the interfacial (bi)carbonate species and show how this non-equilibrium contribution to pH influences interfacial speciation of carbonate and bicarbonate in the near-surface region.

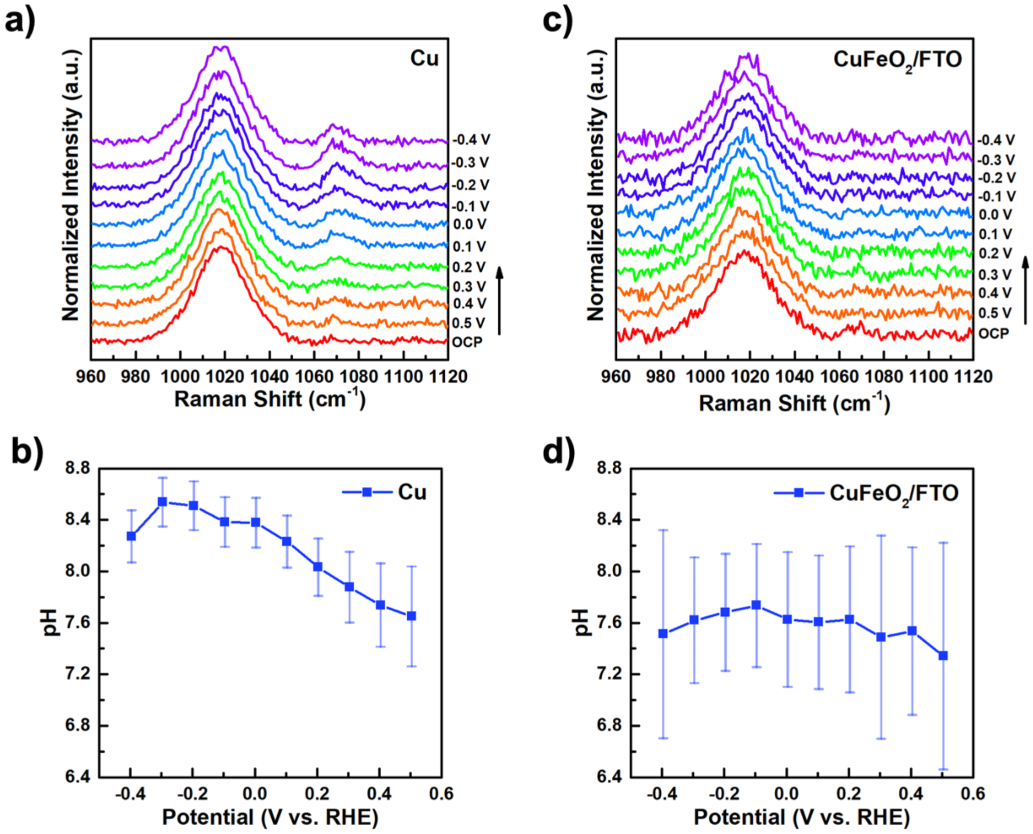

Figure S13. In situ Raman spectra and pHs as a function of applied bias in 1M $NaHCO_3$ on (a-b) Cu and (c-d) $CuFeO_2$/FTO electrodes under CO_2 purging.

S8. In situ VSFG Measurements

The VSFG system used in this work has previously been described in detail.[8] Briefly, the VSFG system uses a femtosecond Ti:Sapphire regenerative amplifier (Spectra Physics - Solstice) which produces 800 nm pulses with a pulse duration of 90 fs and an average power of 7 W at a 2 kHz repetition rate. Then, 70% of the output pulse is used to pump an optical parametric amplifier

(OPA from Topas Prime), which directs the signal and the idler to a noncollinear difference frequency generation (nDFG) stage. This produces a tunable broadband infrared (IR) beam. The remaining 30% of the output pulse is spectrally narrowed to about 10 cm^{-1} using an air-gap etalon (TecOptics). The 800 nm beam goes through a time delay stage so that the time between the 800 nm and IR pulses can be controlled, minimizing χ^3 contributions of the substrate. The experiments are conducted in a Kretschmann reflection geometry, where the incident 800 nm and IR beams are coupled through a CaF_2 prism at a 56 °C incident angle to the surface where the 800 nm and IR beams are spatially and temporally overlapped. The prism is attached to the substrate side of the sample using a perfluorodecane index matching fluid to fill the air gaps between the CaF_2 substrate and prism. The working electrode is placed inside a home-built electrochemical cell, with the working electrode making contact with the electrolyte solution within the cell.

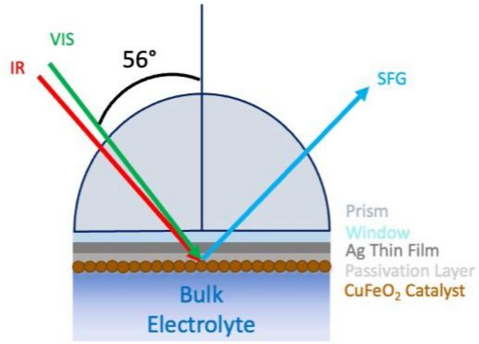

Figure S14. VSFG cell design for in situ measurements.

A schematic of the VSFG sample geometry is given in Figure S14. The reflection of the 800 nm, IR, and SFG signal are collected. The 800 nm and IR beams are filtered out through appropriate pass filters while the SFG beam is focused into a spectrometer (Andor Shamrock 303i) and onto a

CCD detector (Andor Newton DU920P-BVF). All measurements were collected in a ppp polarization (SFG, 800 nm, and IR beams are all p-polarized) collected with 2 min integration.

The configuration of the VSFG technique allows light to couple into the surface metal plasmon modes, providing a plasmon-enhancement effect. We deposit 50 nm of Ag onto the CaF_2 window to use as a substrate for the plasmonic effect. Since CO is easily adsorbed and detected via VSFG to Ag, a thin 2-3 nm layer of Al_2O_3 was deposited on top of the Ag that acts as a passivation layer and prevents CO adsorption. $CuFeO_2$ was drop casted on top of the $Al_2O_3/Ag/CaF_2$ electrode.

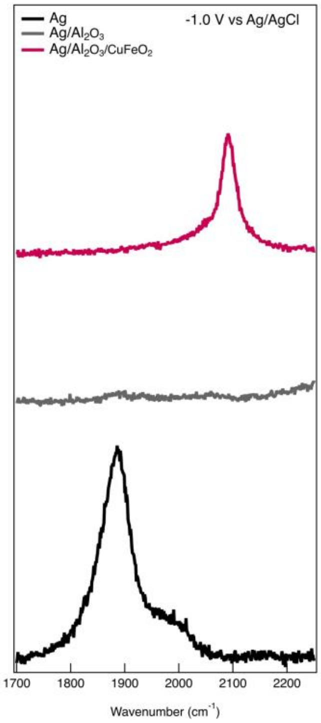

Figure S15. VSFG spectra of CO adsorption under a -1.0 V vs Ag/AgCl bias on Ag (black), Ag/Al_2O_3 (gray), and $Ag/Al_2O_3/CuFeO_2$ (red) in CO-saturated 0.05 M Na_2CO_3.

Figure S15 illustrates the effects of the Al_2O_3 passivation layer used here. Bare Ag deposited on CaF_2 shows strong CO adsorption in CO-purged Na_2CO_3 at a fixed -1.0 V vs Ag/AgCl, with a peak at 1887 cm^{-1} due to surface-adsorbed CO. The addition of the Al_2O_3 passivation layer shows no CO adsorption to the surface under identical experimental conditions. After drop casting

$CuFeO_2$ on top, CO is adsorbed to the surface at 2091 cm^{-1}, distinctly different than the CO adsorption to bare Ag. This shows that the CO adsorption measured in the main manuscript is due to CO adsorption to the $CuFeO_2$ film and not the underlying Ag substrate used for plasmon-enhancement in these measurements.

To prove that the signals seen in the main manuscript and also in Figure S15 originate from surface-adsorbed CO, we repeat the VSFG measurement in N_2 purging. Figure S16 shows that without the presence of CO, no spectral feature is seen.

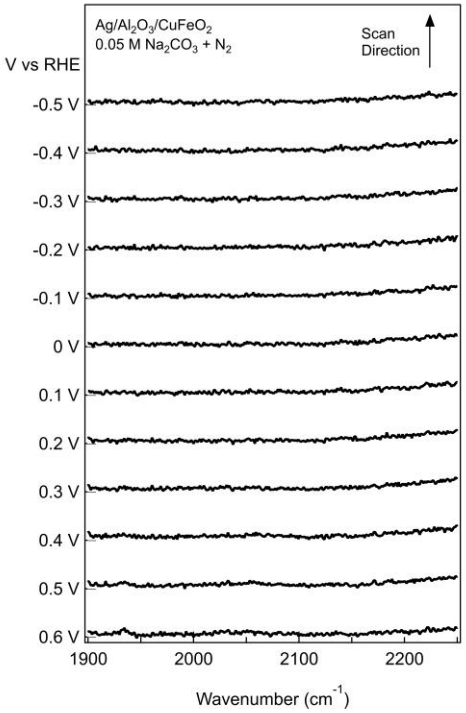

Figure S16. VSFG spectra of N_2-purged 0.05 M Na_2CO_3 with a sweeping applied bias.

References

(1) Elkhouni, T.; Amami, M.; Colin, C. V.; Salah, A. B. Structural and Magnetoelectric Interactions of (Ca, Mg)-Doped Polycrystalline Multiferroic $CuFeO_2$. *Materials Research Bulletin* **2014**, *53*, 151–157.

(2) Aktas, O.; Truong, K. D.; Otani, T.; Balakrishnan, G.; Clouter, M. J.; Kimura, T.; Quirion, G. Raman Scattering Study of Delafossite Magnetoelectric Multiferroic Compounds: $CuFeO_2$ and $CuCrO_2$. *Journal of Physics: Condensed Matter* **2012**, *24* (3), 036003.

(3) Xu, J. F.; Ji, W.; Shen, Z. X.; Li, W. S.; Tang, S. H.; Ye, X. R.; Jia, D. Z.; Xin, X. Q. Raman Spectra of CuO Nanocrystals. *Journal of Raman Spectroscopy* **1999**, *30* (5), 413–415.

(4) Fondell, M.; Gorgoi, M.; Boman, M.; Lindblad, A. An HAXPES Study of Sn, SnS, SnO and SnO_2. *Journal of Electron Spectroscopy and Related Phenomena* **2014**, *195*, 195–199.

(5) Zhao, X.; Wen, T.; Zhang, J.; Ye, J.; Ma, Z.; Yuan, H.; Ye, X.; Wang, Y. Fe-Doped SnO_2 Catalysts with Both BA and LA Sites: Facile Preparation and Biomass Carbohydrates Conversion to Methyl Lactate MLA. *RSC Advances* **2017**, *7* (35), 21678–21685.

(6) Nasir, M.; Khan, M.; Rini, E. G.; Agbo, S. A.; Sen, S. Exploring the Role of Fe Substitution on Electronic, Structural, and Magnetic Properties of La_2NiMnO_6 Double Perovskites. *Applied Physics A* **2021**, *127* (3), 208.

(7) Zhang, Z.; Melo, L.; Jansonius, R. P.; Habibzadeh, F.; Grant, E. R.; Berlinguette, C. P. pH Matters When Reducing CO_2 in an Electrochemical Flow Cell. *ACS Energy Letters* **2020**, *5* (10), 3101–3107.

(8) Wallentine, S.; Bandaranayake, S.; Biswas, S.; Baker, L. R. Plasmon-Resonant Vibrational Sum Frequency Generation of Electrochemical Interfaces: Direct Observation of Carbon Dioxide Electroreduction on Gold. *Journal of Physical Chemistry A* **2020**, *124* (39), 8057–8064.
